# Real‐Time Monitoring of Mass and Mechanical Properties in Single Cells and Multicellular Spheroids

**DOI:** 10.1002/advs.77926

**Published:** 2026-09-27

**Authors:** Ilaria Incaviglia, Giulia E. M. Ammirati, Sophie Herzog, Tommy Krug, Nico Strohmeyer, Miguel Camacho Rufino, Matthias P. Lutolf, David A. Weitz, Daniel J. Müller

**Affiliations:** ^1^ Department of Biosystems Science and Engineering ETH Zürich Basel Switzerland; ^2^ John A. Paulson School of Engineering and Applied Sciences Harvard University Cambridge Massachusetts USA; ^3^ Institute of Human Biology (IHB) Roche Pharma Research and Early Development (pRED), Roche Innovation Center Basel, F. Hoffmann‐La Roche Ltd, Basel Basel Switzerland

**Keywords:** cancer, cell mass, cell mechanics, cell state, human fibroblasts, human spheroids, mechanobiology, microcantilever, mouse fibroblasts, quality‐factor

## Abstract

Cell state, growth, and mechanics are tightly connected properties underpinned by fundamental biological processes. Yet methods to quantify them simultaneously under physiologically relevant conditions, at resolutions sufficient to follow (sub)cellular processes, are still lacking. In particular, the correlation between cell state, mass, and mechanical properties during dynamic processes such as growth, differentiation, development, and disease remains poorly understood. Here, we develop experimental and theoretical approaches based on photothermally actuated microcantilevers integrated with optical microscopy to simultaneously monitor the morphology, mass, and quality (Q)‐factor of single mammalian cells and larger cellular systems under culture conditions, with millisecond time resolution. We find that the Q‐factor, a measure of mechanical energy dissipation, mirrors the mechanical properties of the cell, with the cytoplasm dominating as the largest and softest compartment. Our method reveals how cellular mechanical properties correlate with mass and depend on cell type, state, and growth. Applied to human spheroids, it further monitors how larger cellular systems regulate mechanical properties during development. Together, these measurements quantify mechanobiological parameters that have so far remained inaccessible, opening the way to a more complete characterization of how cellular systems regulate mechanics during fundamental processes of life.

## Graphical abstract

This study introduces photothermally actuated microcantilevers combined with optical microscopy to simultaneously measure morphology, mass, and mechanical properties of cellular systems in real‐time. The findings link cytoplasm‐dominated mechanical energy dissipation to the Q‐factor as a novel biophysical parameter. The theoretical and experimental approach offers unique insight into how single cells and larger cellular systems regulate growth, differentiation, and mechanical properties. Image copyright Martin Oeggerli, micronaut.ch, and Giulia E.M. Ammirati, ETH Zurich.

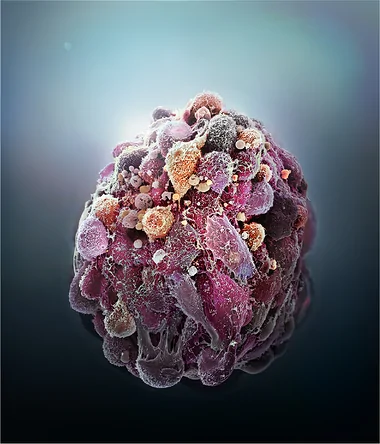

## 1 Introduction

Living cellular systems operate through complex molecular and cellular interactions that collectively translate into specific mechanical properties. Proteins, membranes, condensates, compartments, cells, and the extracellular environment exhibit distinct mechanical properties that dynamically adapt to cellular conditions such as cell state, growth, differentiation, assembly, and homeostasis. Cellular systems gauge these properties at various length and time scales, to alter both individual and collective responses [1, 2, 3, 4]. For example, most mammalian cells progress through mitosis by actively rounding against external mechanical constraints of the tissue into a spherical shape [5, 6, 7]. If rounding mitotic cells cannot create sufficient space, their genetic material will be asymmetrically distributed, followed by malfunction and cell death [8]. On a larger scale, mechanical cues within cellular systems are essential for the proper development and function of tissues, organoids [9, 10, 11], and organs [12, 13]. Thus, quantifying the mechanical properties and responses of cells is essential to understand how they contribute to fundamental cellular processes, disease development, and potential therapeutic interventions.

Exciting technologies have been established to measure the mechanical properties of cells and complex cellular systems, such as tissues, organoids, or organs. Among them are atomic force microscopy (AFM) [14], optical [15] and magnetic [16] tweezers, droplets [17, 18], traction force microscopy [19, 20], or micropipette aspiration [21]. In most applications, these approaches focus on (sub)cellular measurements of the mechanical properties of the actomyosin cortex, filaments, cell adhesion, traction, tension, or stress. Thus, they address local or specific aspects of cellular mechanics. However, the question remains as to what comprehensively describes the mechanical properties of an entire cell. This understanding holds significant physiological relevance, as cells are heterogeneous in structure, state, and mechanical properties. Hence, probing these properties locally or only in parts may only reveal fractions of the mechanical landscape a cell processes for making decisions. Although the mechanical properties and response of single cells influence how they integrate into complex cellular systems and how these systems respond, they depend on the mass or volume of the cell [22, 23]. Moreover, instead of characterizing the mechanical properties of a cell at a certain time point, it is important to be able to continuously monitor the mechanical properties of cells and cellular systems and link them to the cell state (or process) during which they change. However, how cell mass, mechanical properties, and state correlate in real‐time has been difficult to monitor.

The quality (Q)‐factor is widely used to describe mechanical damping and defined as the ratio of mechanical energy stored to the energy dissipated in physical oscillators such as microcantilevers [24]. Ultrasensitive optomechanical disk resonators were recently employed to measure the Q‐factor of single bacteria dehydrated in vacuum and air [25]. To describe more biologically relevant scenarios, photothermally actuated resonating microcantilevers have been introduced to measure the total mass of single cells under culture conditions, while observing morphology by optical microscopy [26, 27, 28]. Recently, multiplexing of cell morphology, mass, and resonance frequency allowed an approximation of the Q‐factor of living mammalian cells at the population level, which is of particular interest because cell state, mass, and mechanics are tightly interlinked [29]. Similarly, microcantilevers have been used to approximate the resonance frequency and higher vibrational modes of mammalian cells [30]. However, although the latter two studies approached the Q‐factor of mammalian cells, they could not conclude which cell mechanical properties the Q‐factor describes and could not measure these properties at sufficient resolution such as needed to monitor dynamic cellular processes or states. To date, no technologies are available that can continuously monitor the Q‐factor of single living mammalian cells or larger cellular systems at sufficient time and mass resolution to allow following cellular processes in real‐time.

Here, we introduce the experimental and theoretical approaches to apply photothermally actuated microcantilevers in combination with optical microscopy to continuously monitor the morphology, mass, and Q‐factor of mammalian cells under culture conditions, at millisecond time resolution. We show that the Q‐factor predominantly describes the mechanical energy dissipation of the cellular cytoplasm, which is the largest and possibly the softest cellular compartment [14, 31]. By applying our nanotechnological approach to various mammalian cell types, we reveal unique insights into the mechanical properties that cells exhibit during growth and how their mechanical properties depend on cell type, cellular components, and state. Furthermore, we monitor for the first time the mass of larger cellular systems, such as human spheroids, and reveal how they regulate mechanical properties during growth. These results open a unique avenue into mechanobiology, such as how cellular systems sense, regulate, and apply mechanical properties during basic cellular processes.

## 2 Using Microcantilevers to Monitor the Q‐Factor of a Cell

**FIGURE 1:**
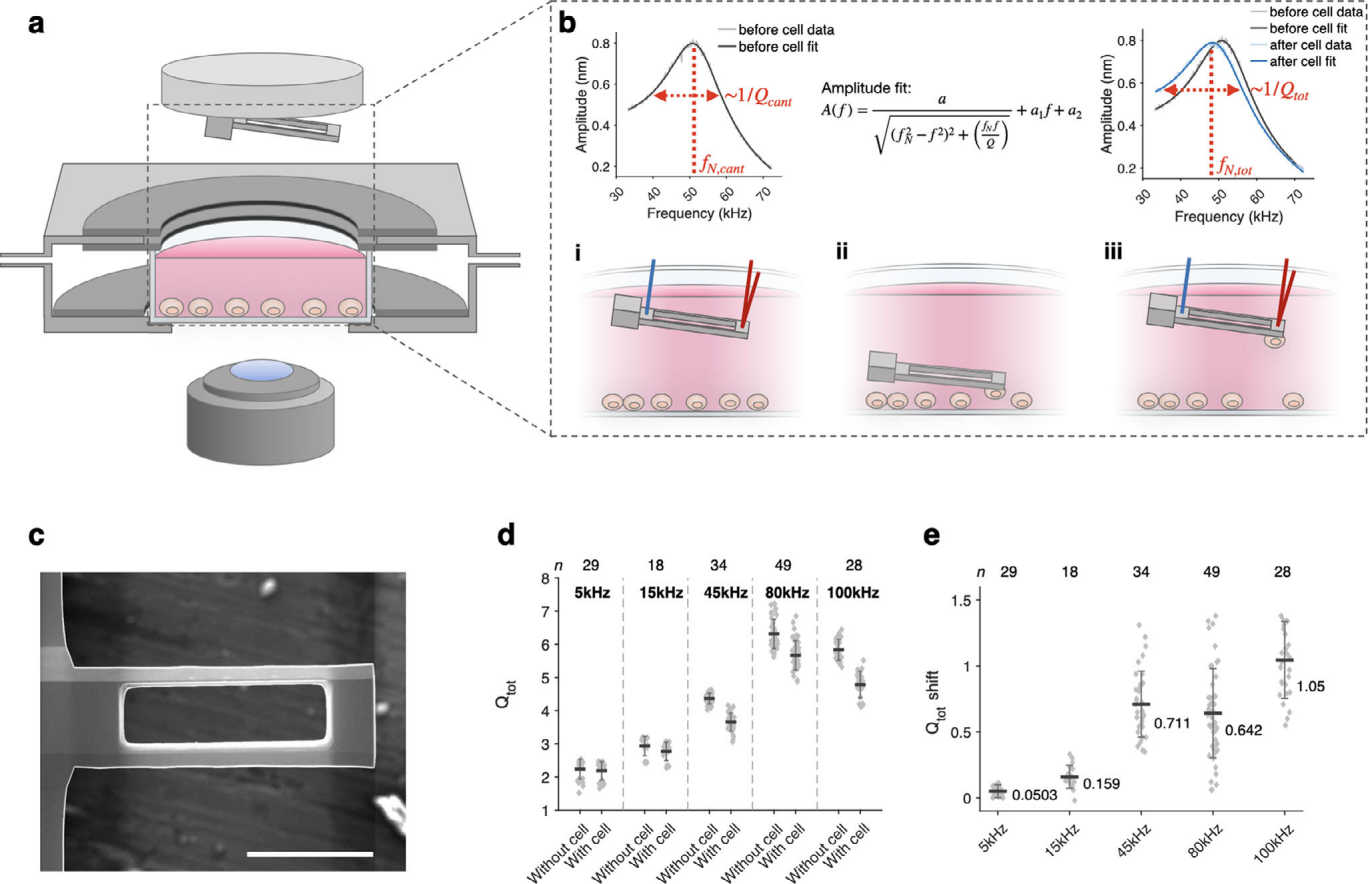
Working principle of the picobalance with resonance frequency and Q‐factor extraction. (a) The inertial picobalance encompasses an environmental chamber that surrounds the microcantilever and cells. The chamber controls temperature, gas composition, and humidity to mitigate culture medium evaporation and ensure cell culture conditions [37]. The setup includes optical microscopy to monitor cell morphology and state. (b) Representative frequency sweeps and measurement protocol: (i) A low‐powered infrared laser (852 nm) detects the deflection of the free end of the microcantilever. The amplitude of the oscillating cantilever can be tracked through sweeping the frequency of the actuating laser, and the resonance frequency *f_N_* and Q‐factor *Q_cant_* of the cantilever can be extracted by fitting the amplitude with the following model: $A\left(f\right)=\frac{a}{\sqrt{{\left(f_{N}^{2}\;-\;f^{2}\right)}^{2}+{\left(\frac{f_{N}\cdot f}{Q}\right)}^{2}}}+a_{1}\cdot f+a_{2}$ (Equation 3). (ii) After measuring *f*_*N*,*cant*_ and *Q_cant_*, the lasers are turned off and the cantilever is lowered to contact a target cell to establish attachment. (iii) The cantilever is then retracted to its original position, and another frequency sweep is recorded to extract *f*_*N*,*tot*_ and *Q_tot_*. (c) SEM top view of the bare microcantilever, which had been FIB‐sculpted to enhance its sensitivity for mass measurements of single mammalian cells [28]. Scale bar, 50 µm. d, *Q_tot_* of ConA‐coated cantilevers measured before and after HeLa cell attachment depend on *f*_*N*,*cant*_. Measurements were performed under cell culture conditions (37°C and 5% CO_2_) in DMEM medium supplemented with FBS following overnight starvation of the cells. *f*_*N*,*cant*_ of the cantilevers were categorized into 5, 15, 45, 80, and 100 kHz. (e) The shift in *Q_tot_* upon cell attachment increases with *f*_*N*,*cant*_ and is highest for the cantilevers with *f*_*N*,*cant*_ ≈ 100 kHz. Data are presented as the mean ± *s.d*., and *n* indicates the number of independent cells measured. A maximum of 3 cells were measured for each cantilever used. Extended mean and *s.d*. values are shown in Figure S1a.

Originally, the picobalance was introduced to non‐invasively measure the inertial (or total) mass of adherent cells at high mass and time resolution [27]. Mounted on an inverted optical microscope and operated under cell culture conditions, the picobalance uses silicon microcantilevers as mass sensors (Figure 1a). The base of the microcantilever is photothermally actuated by either a blue (405 nm) or infrared (785 nm) laser [26, 27, 28, 32] to oscillate the free end of the microcantilever at amplitudes of ≈ 1–15 Å. Attaching a cell to the free end of the microcantilever changes the mass of the resonating system and thus the resonance frequency *f_N_* of the cantilever, which is continuously tracked via frequency sweeps (Figure 1b). Monitoring *f_N_* before and after attachment of the cell determines the resonance frequency shift Δ*f_N_*, which can be converted into the mass Δ*m_cell_* of the cell attached to the microcantilever by $\Delta m_{cell}=\frac{k}{4\pi^{2}}\cdot \left(\frac{1}{{\left(f_{N}\;-\;\Delta f_{N}\right)}^{2}}\;-\;\frac{1}{f_{N}^{2}}\right)$ (Equation 1) [33], where *k* is the spring constant of the cantilever [27]. Here, we extend this technology to simultaneously monitor both resonance frequency and Q‐factor shift induced by cell attachment. The Q‐factor represents the ratio of energy stored in the resonating microcantilever vs. the energy loss per oscillatory cycle [24]. When a non‐point mass analyte, such as a micrometer‐sized cell, attaches to the oscillating cantilever, the Q‐factor shows two opposing shifts: *i*) an increase due to the added cell mass [34], and *ii*) a decrease resulting from the additional mechanical energy loss caused by the non‐rigid cell [35]. We combined both effects into one equation to describe the total Q‐factor of the cantilever and cell system $\frac{1}{Q_{tot}}=\frac{1}{Q_{cant}}+\frac{1}{Q_{cell}}\;-\;\frac{1}{Q_{mass}}$ (Equation 2), where *Q_cell_* reflects the ratio of the mechanical energy dissipated by the non‐rigid cell body and *Q_mass_* the ratio of the mechanical energy stored by the cell mass (Methods). To optimize both mass and Q‐factor sensitivity for single cell measurements, we tailored the cantilever shape using a focused ion beam (FIB) to reduce its inertial mass [28] and conducted frequency sweeps to determine *f_N_* (Figure 1b,c). Afterwards, we attached single rounded HeLa cells to the cantilevers with different resonance frequencies and monitored the Q‐factor changes (Figure 1d,e and Figure S1). The different resonance frequencies compared were obtained using cantilevers of the same material, thickness, and width but of different lengths, tailored by FIB milling. Because *f_N_* of a rectangular cantilever scale inversely with the square of its length, *f_N_* and geometry necessarily co‐vary. The pronounced increase of the Q‐factor shifts with *f_N_* nevertheless reflects the resonance frequency itself, as at higher *f_N_* the oscillation period approaches the viscoelastic relaxation times of the cell and a larger fraction of the mechanical energy is dissipated per cycle by the non‐rigid cell body [36]. Microcantilevers with *f_N_* of ≥ 45 kHz showed much more pronounced Q‐factor shifts upon cell attachment, thus highlighting their higher sensitivity for detecting Q‐factor changes. However, previous cell mass measurements using resonating microcantilevers show that they provide better mass sensitivity at lower resonance frequencies [28]. Thus, as a deliberate compromise to best measure Q‐factor and cell mass at high sensitivity, we choose cantilevers with *f_N_* ≈ 45 kHz for our experiments.

## 3 Q‐Factor Depends on Cell Stiffness Independent of Cell Mass

**FIGURE 2:**
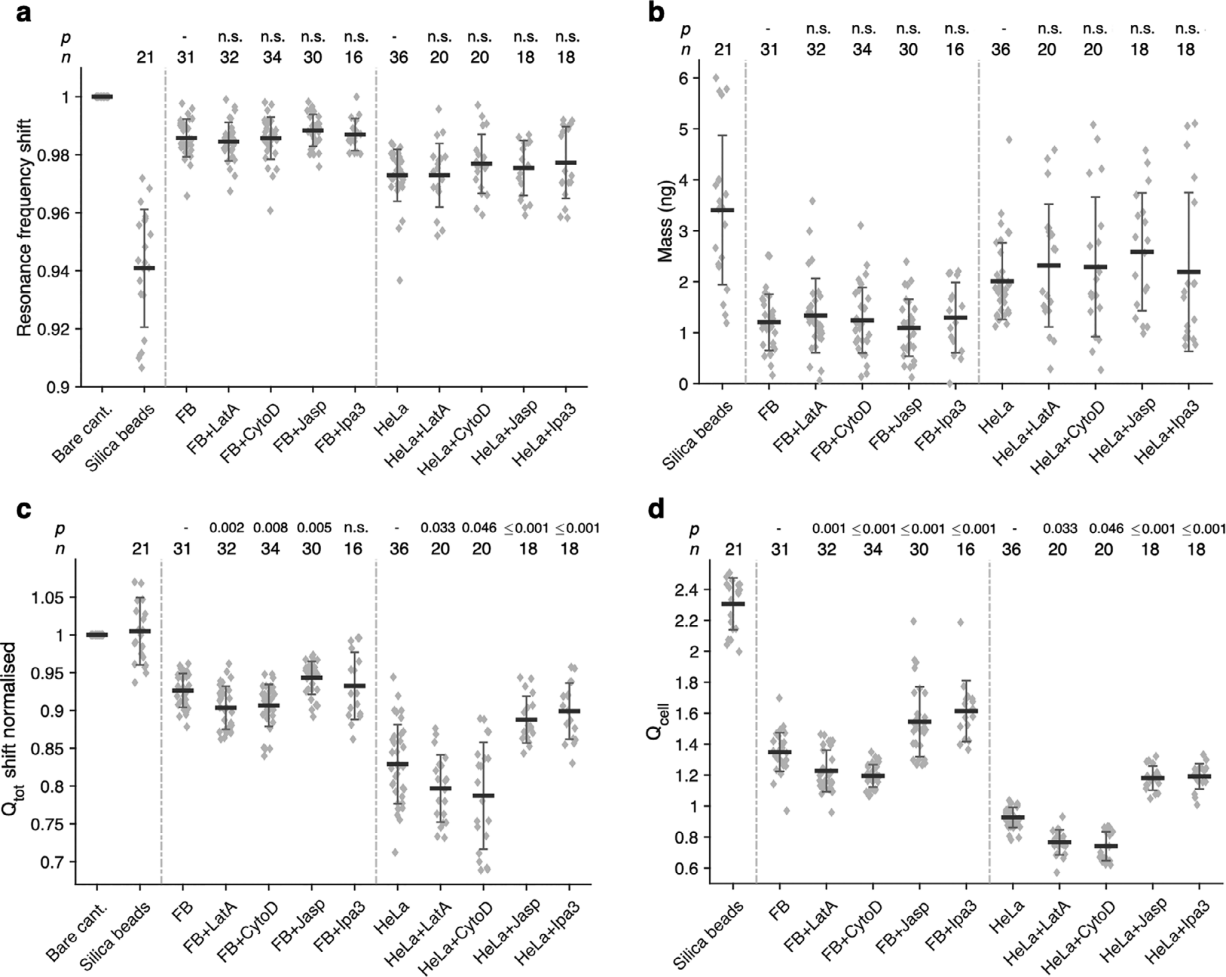
Resonance frequency *f_N_* and Q‐factor of animal cells follow perturbations in cell stiffness. (a) Resonance frequency shift Δ*f_N_* of microcantilevers induced by the attachment of 15 µm diameter silica beads, fibroblasts (FB), or HeLa cells. Living cells were measured in the unperturbed state with DMSO as the control vehicle and after perturbing their actomyosin cortex with latrunculin A (LatA), cytochalasin D (CytoD), jasplakinolide (Jasp), or IPA3. (b) Mass of silica beads, HeLa cells, and fibroblasts derived from Δ*f_N_*. The inertial mass was corrected for the position of the bead or cell along the microcantilever (Methods). (c) Normalized *Q_tot_* shift following bead or cell attachment to the microcantilever. Here, the normalized shift is defined as the Q‐factor change of *Q_tot_* upon attachment of a bead or cell to the cantilever divided by the Q‐factor of the bare cantilever measured immediately before attachment. (d) *Q_cell_* of attached beads and cells, derived from *Q_tot_* as described in Equation 2 (Equation 7 in Methods). Data are presented as mean ± *s.d*., with *n* indicating the number of independent beads or cells analyzed. Statistical significance was evaluated using the Wilcoxon rank sum test. *f_N_*, inertial mass, Δ*Q_tot_*, and *Q_cell_* of wild‐type mouse fibroblasts and HeLa cells were monitored 5 min after attachment to the microcantilever. To perturb the cytoskeleton, cells were preincubated for 30 min before measurements with either 20 µm DMSO (control), 5 µm latrunculin A (LatA), 5 µm cytochalasin D (CytoD), 5 µm jasplakinolide (Jasp), or 20 µm IPA3. All measurements were conducted in DMEM medium without FBS (starving conditions) at 37°C with 5% CO_2_ using ConA‐coated cantilevers. *P* values are given in figures, values >0.05 are considered non‐significant (n.s.).

To investigate the impact of cell stiffness on the Q‐factor, we measured the resonance frequency shift Δ*f_N_*, mass, and Q‐factor shift resulting from the attachment of single silica beads, mouse fibroblasts, or HeLa cells to ConA‐coated cantilevers (Figure 2a–c). The starved cells were characterized in the unperturbed state and after treatment with the F‐actin destabilizers latrunculin A (LatA) or cytochalasin D (CytoD), the F‐actin stabilizer jasplakinolide (Jasp), or the p21‐activated kinase 1 (PAK1) inhibitor IPA3. The attachment of silica beads changed *f_N_* of the microcantilever by ≈ 6%, while the attachment of living cells changed *f_N_* by ≲ 3% (Figure 2a). These frequency changes correspond to position‐corrected masses (Methods) of 3.41 ± 1.46 ng (mean ± s.d.) for silica beads, 1.20 ± 0.56 ng for fibroblasts, and 2.0 ± 0.75 ng for HeLa cells in the unperturbed state (Figure 2b). Notably, after cytoskeletal perturbation the cell mass did not change significantly. Simultaneous measurement of the Q‐factor of the entire system of cantilever and cell *Q_tot_* revealed distinct Q‐factor shifts (Figure 2c). For both fibroblasts and HeLa cells, the cytoskeletal inhibitors LatA and CytoD considerably decreased *Q_tot_*. In contrast, treating HeLa cells with Jasp or IPA3 increased *Q_tot_*, and treating fibroblasts with Jasp or IPA3 increased *Q_tot_*. The attachment of silica beads to the cantilever barely changed *Q_tot_*, which is likely because the stiffness of beads matched that of the cantilever, which are of the same material, thus leading to minimal mechanical energy dissipation.

To extract the Q‐factor of the cell *Q_cell_* from *Q_tot_*, we derived an equation accounting for the positive Q‐factor shift caused by the added mass of the adhering cell, which is predicted using the cell mass values calculated from *f_N_* shifts, and the negative Q‐factor shift arising from the mechanical properties of the cell (Equation 2, Methods). Applying this equation revealed that the *Q_cell_* of wild‐type fibroblasts decreased from 1.35 ± 0.12 to 1.23 ± 0.13 in the presence of LatA and to 1.19 ± 0.08 in the presence of CytoD (Figure 2d). Conversely, incubation with Jasp or IPA3 increased *Q_cell_* to 1.54 ± 0.23 or 1.61 ± 0.2, respectively. HeLa cells in the unperturbed state showed a lower *Q_cell_* of 0.93 ± 0.03. The consistently lower *Q_cell_* values of HeLa cells align well with previous reports indicating that HeLa cells tend to have softer viscoelastic properties than fibroblasts [38, 39]. Destabilization of F‐actin in HeLa cells with LatA or CytoD reduced *Q_cell_* to 0.76 ± 0.09 or 0.74 ± 0.09, respectively, while stabilization with Jasp or treatment with IPA3 increased *Q_cell_* to 1.18 ± 0.08 or 1.19 ± 0.08.

The elevated *Q_cell_* values extracted for silica beads relative to cells have a simple physical origin (Figure 2d). Silica is a nearly ideal elastic solid with very low internal friction, and its Young's modulus (≈70 GPa) is of the same order as that of the silicon cantilever (≈170 GPa) [40]. A body this much stiffer than a cell (whose cytoplasmic modulus is ≈10^2^ Pa and cortical modulus ≈10^3^–10^4^ Pa [14, 31]) does not deform relative to the cantilever and co‐oscillates as a rigid mass, adding inertia without dissipating measurable energy, so that *Q_cell_* approaches the near‐loss‐free limit. Living cells, in contrast, are soft and viscoelastic; under oscillation their body deforms and dissipate a substantial fraction of the stored energy each cycle, strongly lowering *Q_cell_*.

Our findings indicate that while cell mass measurements remain insensitive to changes in cytoskeletal stiffness caused by (de‐)stabilizing the actomyosin cortex, these perturbations affect the Q‐factor of the cell. Thus, our experimental setup, which detects cell mass and Q‐factor independently of each other, shows that both parameters are not necessarily linked in living cells. Since actin filaments and myosin are present in the cortex and across the cytoplasm of the cell [41, 42, 43], the perturbation of actin and myosin also affects the mechanical properties of the cytoplasm, which is the largest compartment of the living cell. Hence, the *Q_cell_* changes observed reflect a global remodeling of the cytoplasmic actin network rather than a cortex‐specific effect. Next, we thus wanted to characterize whether *Q_cell_* best describes the mechanical properties of cortex or cytoplasm, or both.

## 4 Q‐Factor is Largely Independent of Cell Growth and Cortical Stiffness

The acute pharmacological perturbations applied in the preceding section alter the actin cytoskeleton non‐specifically. We thus searched for cell lines that provide a genetically defined and non‐pharmacological means of tuning cytoskeletal organization throughout the cell. Cell stiffness, however, also relates to cell adhesion, which is established differently through the various members of cell adhesion molecules such as the prominently represented integrin family [44, 45]. Integrins are transmembrane receptors, many of which recognize the arginine–glycine–aspartate (RGD) motif present in extracellular matrix ligands. Among these RGD‐binding integrins, β1‐class integrins, including α5β1 and α8β1 integrins, mediate high‐affinity adhesion to fibronectin and nephronectin, respectively [46, 47, 48, 49]. For α5β1 integrin, binding is governed by the RGD motif and the adjacent synergy site PHSRN of fibronectin [50], which markedly increases the binding affinity, strength, and stability of cell adhesion, and the resulting force transmission and downstream signaling [51]. α8β1 integrin binds RGD‐containing ligands including nephronectin and fibronectin, contributing to adhesion in a tissue‐ and context‐dependent manner. Alongside β1‐class integrins, αV‐class integrins act as a second, parallel set of fibronectin receptors through which αVβ3, αVβ5, αVβ6 (and αVβ1) integrins recognize the RGD site in fibronectin [52, 53].

**FIGURE 3:**
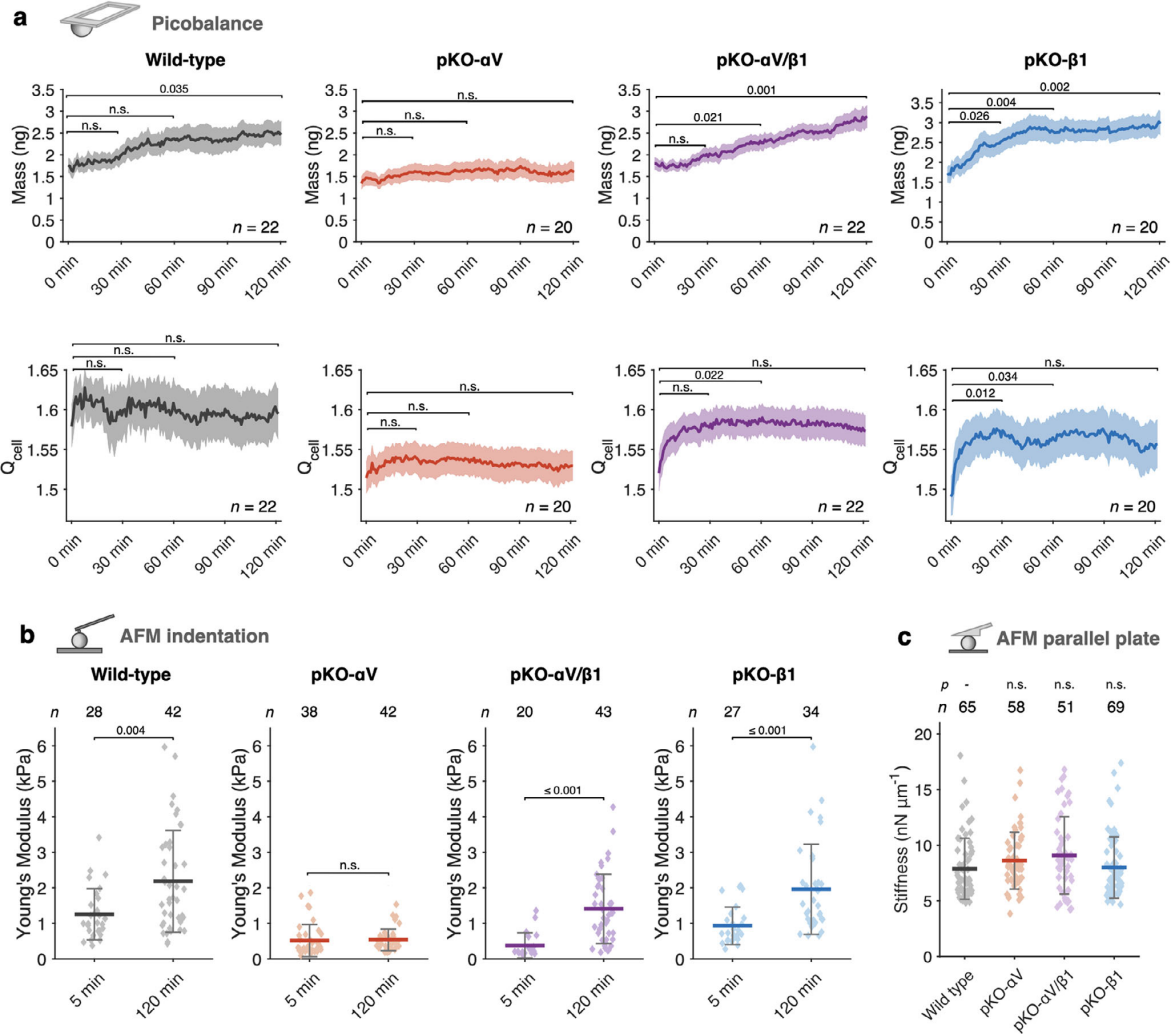
Mechanical profiling of integrin knockout fibroblast cell lines under physiological conditions. (a) Mass and Q‐factor of wild‐type, pKO‐αV, pKO‐αV/β1 and pKO‐β1 fibroblasts adhering to fibronectin‐coated cantilevers and monitored for 2 h. Measurements were performed under cell culture conditions (37°C and 5% CO_2_) in DMEM medium supplemented with FBS following overnight starvation. (b) Actomyosin cortex stiffness of the fibroblasts was probed using tipless AFM cantilevers with 5 µm diameter silica beads glued to their free end. Cells were probed while adhering to fibronectin‐coated Petri dishes following a 5–10 min adhesion period in DMEM medium supplemented with FBS at 37°C. (c) Stiffness of rounded fibroblasts as derived through a parallel plate assay using wedged microcantilevers [8]. Wedged microcantilevers were approached to compress single rounded cells at a speed of 5 µm s^−1^ until reaching 25 nN. The experiments were conducted in DMEM medium supplemented with FBS at 37°C. The apparent contact stiffness was determined from the slope of the approach force‐distance curve between 5 and 20 nN [55]. Data in (a‐c) are presented as mean ± *s.d*. with *n* indicating the number of independent cells measured. Statistical significance was evaluated using the Wilcoxon rank sum test. *P* values are given in figures, values >0.05 are considered non‐significant (n.s.).

To characterize whether differences in establishing adhesion and thus in stiffening the cell cytoskeleton affect the Q‐factor and mass, we monitored both parameters of various αV‐ and β1‐class integrin knockout fibroblast cell lines adhering to fibronectin (Figure 3a). Wild‐type fibroblasts and pan‐knockout (pKO) fibroblasts, in which αV, β1, or both integrin subunits were reintroduced (pKO‐αV, pKO‐β1, and pKO‐αV/β1) [49] were monitored over 2 h as they initiated and strengthened adhesion to fibronectin‐coated cantilevers. Because the pKO fibroblast lines derive from the same parental mouse embryonic fibroblast [49], they are closely matched in cell type, size, and molecular composition, so that the integrin subtype each line expresses is the controlled variable. The distinct integrin repertoires give rise to different cytoskeletal organizations (Figure S2) and, in turn, to different mechanical properties, allowing us to disentangle cortical from cytoplasmic contributions to *Q_cell_*.

Wild‐type, pKO‐αV/β1, and pKO‐β1 fibroblasts considerably increased mass during this timeframe, whereas pKO‐αV fibroblasts showed relatively stable mass. The mass increase observed for wild‐type and pKO‐αV/β1 fibroblasts closely relates to changes in cell volume estimated by confocal microscopy (Figure S3). Furthermore, wild‐type and pKO‐β1 fibroblasts increased mass within 30 min and thereafter showed roughly stable mass, thus suggesting a growth pause [54], while pKO‐αV/β1 fibroblasts grew throughout the time course of the measurement. The continuous measurement of *Q_cell_* showed a different trend compared to cell mass, and after a relatively short attachment time of ≈ 5 min reached stable values around which the cells fluctuated throughout the measurement. Besides these features, which were common for all fibroblast lines, the mass and Q‐factor curves showed trends specific to individual fibroblast lines, thus highlighting distinct integrin‐dependent growth and mechanical properties.

To characterize to what extent the actomyosin cortex contributes to *Q_cell_* of the different fibroblast lines and perturbations, we conducted two sets of AFM experiments to measure cortex stiffness. In the first set, beads of 5 µm diameter were glued to AFM cantilevers and used to indent fibroblasts 5 min and 2 h after they initiated adhesion to fibronectin‐coated Petri dishes (Figure 3b). Thereby, an indentation depth into fibroblasts of ≈ 800 nm was analyzed to primarily detect the mechanical properties of the actomyosin cortex [14]. Force‐distance curves recorded during fibroblast indentation allowed extraction of the Young's modulus of the actomyosin cortex [7]. Wild‐type fibroblasts increased the cortical Young's modulus from 1.25 ± 0.72 kPa (mean ± *s.d*.) at 5 min to 2.18 ± 1.43 kPa at 2 h, pKO‐αV/β1 fibroblasts from 0.38 ± 0.34 kPa to 1.40 ± 0.97 kPa, and pKO‐β1 fibroblasts from 0.92 ± 0.52 kPa to 1.95 ± 1.26 kPa. In contrast, the mechanical properties of the cortex of pKO‐αV fibroblasts remained unchanged, with 0.51 ± 0.45 kPa at 5 min and 0.53 ± 0.30 kPa at 2 h. However, the stiffness changes observed in wild‐type, pKO‐αV/β1, and pKO‐β1 fibroblasts did not correlate with their *Q_cell_* trends within 2 h of adhesion. In a second set of experiments, single rounded fibroblasts were compressed between two parallel plates provided by a wedged cantilever and a fibronectin‐coated Petri dish [55] (Figure 3c). The fibroblast stiffness was determined from the approach force‐distance curve recorded during compression [56]. The assay revealed stiffnesses of 7.88 ± 2.75 nN µm^−1^ (mean ± s.d.) for wild‐type, 8.61 ± 2.56 nN µm^−1^ for pKO‐αV, 9.09 ± 3.48 nN µm^−1^ for pKO‐αV/β1, and 7.99 ± 2.77 nN µm^−1^ for pKO‐β1 fibroblasts. However, the differences among the cell lines were not statistically significant. This result contrasts with the variations in *Q_cell_* measured for cells in the rounded state immediately after attachment to the microcantilever (Figure S4).

In summary, the experiments show that fibroblasts grow differently depending on which integrin they express and employ to adhere to fibronectin. The different fibroblast lines show different *Q_cell_* values, which they keep constant after establishing adhesion for > 15 min to the substrate. The *Q_cell_* values, however, do not correlate with the Young's moduli measured for the actomyosin cortex of the fibroblast lines at time points of 5 min or 2 h. Neither do the *Q_cell_* values correlate with the actomyosin cortex stiffness measured for each fibroblast line in parallel plate assays. This suggests that *Q_cell_* is not a reliable indicator for the mechanical properties of the actomyosin cortex.

## 5 Cytoplasm Mechanics Dominates Cellular Q‐Factor

**FIGURE 4:**
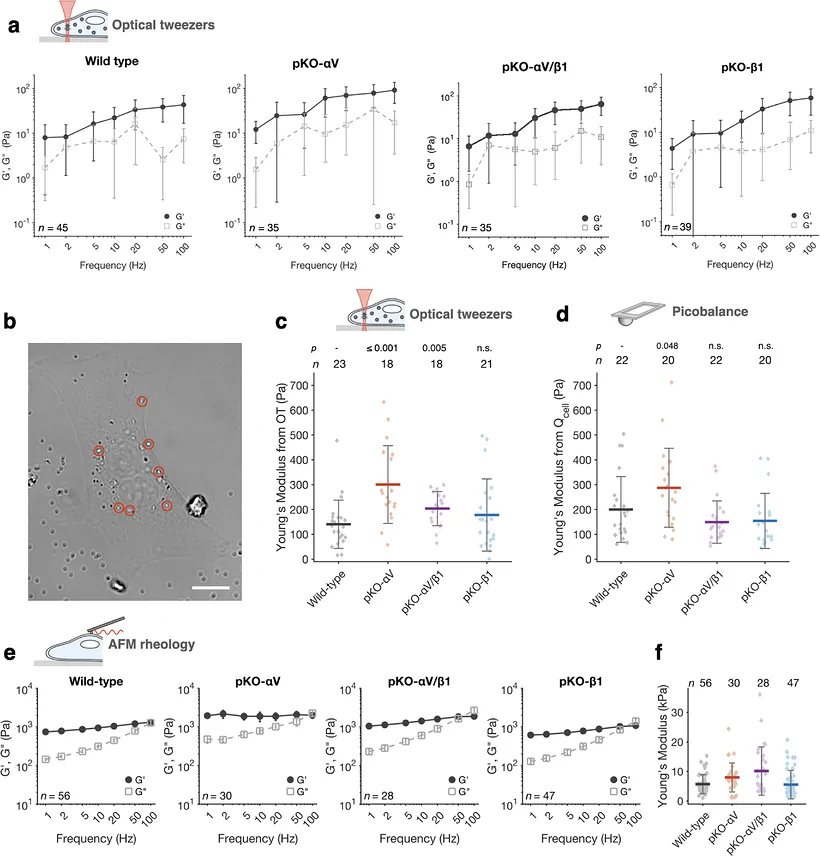
Cytoplasmic Young's moduli extracted from optical tweezers and Q‐factor measurements are of the same order of magnitude. (a) Cytoplasmic stiffness of wild‐type, pKO‐αV, pKO‐αV/β1 and pKO‐β1 fibroblasts as measured using optical tweezers. The frequency‐dependent cytoplasmic storage moduli *G*′ (solid circles) and loss moduli *G*″ (open squares) are determined by assessing the resultant displacement of the bead within the trap oscillation. Data are presented as the mean ± *s.d*., and *n* indicates the number of beads probed. Carboxyl latex beads (0.49 µm in diameter) were internalized by the fibroblasts overnight. To trap and manipulate the fluorescent beads within the cytoplasm, a variable‐power laser was focused through a high‐numerical aperture objective. Stable trapping occurs when the axial gradient force at the focal point exceeds the scattering force pushing the bead along the direction of light propagation. Once trapped, the beads were oscillated by a sinusoidal optical trap across frequencies from 1 to 100 Hz using acousto‐optic deflectors. Both the laser position and bead displacement were recorded simultaneously as the system generated forces at selected frequencies, including 1, 2, 5, 10, 20, 50, and 100 Hz. (b) Bright field image of a wild‐type fibroblast spread on fibronectin‐coated coverslips with endocytosed latex beads highlighted by red circles. Scale bar, 10 µm. (c) Young's modulus extracted from optical tweezer measurements at 100 Hz. Data are presented as interquartile range (grey vertical bars), median (white circle), and mean (colored horizontal bars). *n (osc.)* indicates the number of oscillatory cycles performed while *n (beads)* indicates the number of beads measured. (d) Young's modulus extracted from *Q_cell_* data (Methods). (e) Frequency‐dependent cortical storage moduli *G*′ and loss moduli *G*″ measured by AFM microrheology using tipless AFM cantilevers with 5 µm diameter silica beads glued to their free end. Cells were probed after adhering to fibronectin‐coated Petri dishes for 2 h in DMEM medium supplemented with FBS at 37°C. (f) Young's modulus extracted from AFM microrheology measurements at 100 Hz. Data are presented as mean ± s.d., and *n (cells)* indicates the number of independent cells measured. Statistical significance was evaluated using the Wilcoxon rank sum test. *P* values are given in figures, values >0.05 are considered non‐significant (n.s.).

The pharmacological perturbations (Figure 2) indicate that *Q*_cell_ tracks the cytoplasm‐wide actomyosin network, while our follow‐up experiments (Figures 3 and 4) show that cortical stiffness alone does not account for *Q*_cell_. Building on this, we next asked whether the cytoplasmic stiffness quantitatively explains the *Q*_cell_ values measured across the fibroblast lines. Given that cytoskeletal networks span the entire cell from the plasma membrane to the nucleus [57, 58] (Figure S2), we sought to characterize the contribution of the cytoplasmic stiffness, which encompasses both the cytoskeleton and other intracellular compartments, to the cell mechanical properties measured by *Q*_cell_. We thus employed optical tweezers to characterize whether the differences in *Q_cell_* observed across the fibroblast lines correspond to the mechanical properties of the cytoplasm (Figure S5). Fibroblasts adhering to fibronectin‐coated Petri dishes were allowed to endocytose 0.49 µm diameter carboxyl latex beads overnight, and checked for uniform distribution of the beads across the cytoplasm (Figure S6a,b). The endocytosis of the beads did not interfere with cell viability, as the cells remained spread and healthy even after two days of bead incubation (Figure S6c). The adherent cells were then characterized using optical tweezers (Figure 4a,b). The trapped beads within each fibroblast line were subjected to a sinusoidal oscillating optical trap, and their displacement was recorded to extract the frequency‐dependent elastic and viscous shear moduli of the cytoplasm [59]. The Young's modulus of the cytoplasm was 140.58 ± 97.08 Pa (mean ± s.d.) for wild‐type fibroblasts, 300.29 ± 156.55 Pa for pKO‐αV fibroblasts, 203.58 ± 70.64 Pa for pKO‐αV/β1 fibroblasts, and 177.78 ± 145.33 Pa for pKO‐β1 fibroblasts.

To compare the results from optical tweezers and microcantilevers, we extracted the Young's moduli from both measurements (Methods). The Young's modulus extracted from Q‐factor measurements yielded fewer values, owing to the lower throughput of the microcantilever‐based single cell technique (Figure 4c,d). The Young's modulus of wild‐type fibroblasts was 200.17 ± 132.43 Pa (mean ± s.d.), for pKO‐αV cells 287.38 ± 158.92 Pa, for pKO‐αV/β1 cells 149.19 ± 85.17 Pa, and for pKO‐β1 cells 154.10 ± 110.80 Pa. Surprisingly, the Young's modulus of the cytoplasm as extracted by optical tweezers at 100 Hz was of the same order of magnitude as the Young's modulus extracted from Q‐factor measurements determined with microcantilevers.

Although the optical tweezers probe the cytoplasm at ≤ 100 Hz while the microcantilever operates at a much higher effective frequency, this comparison remains meaningful. Cell rheology follows a weak power law, G*(*f*) ∼ *f* ^α^, with a small exponent (*α* ≈ 0.1–0.2 for the cytoplasm), so that the moduli change only moderately over the intervening frequency range and, being monotonic, preserve the ranking across cell lines; consistently, the optical‐tweezer moduli approach a plateau within the accessible band. Moreover, the AFM microrheology extending up to 500 Hz shows that the cortical moduli are one to two orders of magnitude higher than the *Q_cell_*‐derived moduli and do not track them, whereas the cytoplasmic moduli do, further supporting the assignment of *Q_cell_* to cytoplasmic mechanics.

To further confirm the negligible contribution of the cortex, we performed AFM microrheology on the four fibroblast lines after 2 h of adhesion to fibronectin‐coated dishes (Figure 4e,f). At 100 Hz, the measured Young's moduli were 5.72 ± 3.20 kPa (mean ± s.d.) for wild‐type fibroblasts, 7.99 ± 3.08 kPa for pKO‐αV fibroblasts, 10.17 ± 8.19 kPa for pKO‐αV/β1 fibroblasts, and 5.52 ± 4.78 kPa for pKO‐β1 fibroblasts. These values are one to two orders of magnitude higher than the moduli extracted from Q‐factor measurements, demonstrating that the cortical stiffness cannot account for the observed variations in *Q_cell_*.

## 6 Monitoring Q‐Factor can Distinguish Metastatic From Healthy Fibroblasts

**FIGURE 5:**
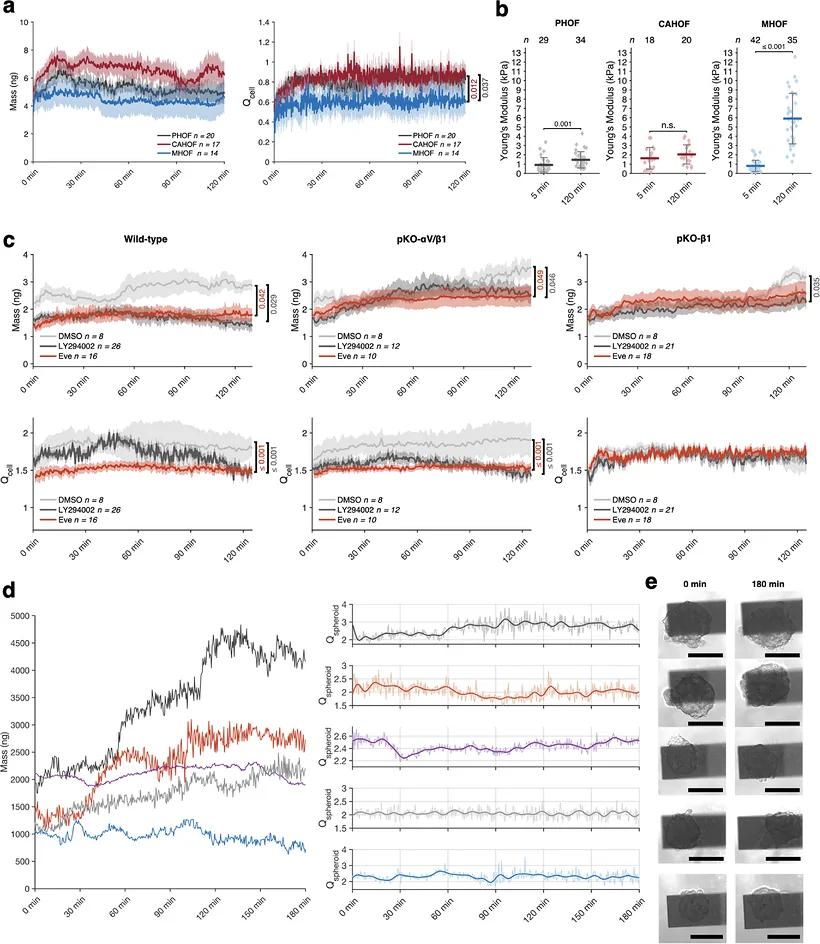
Growth behavior and regulation of mechanical properties of cellular systems. (a) Mass and *Q_cell_* of single primary human ovarian fibroblasts (PHOFs), cancer‐associated human ovarian fibroblasts (CAHOFs) and metastatic human ovarian fibroblasts (MHOFs) adhering to fibronectin‐coated cantilevers under cell culture conditions (37°C and 5% CO_2_) in DMEM medium supplemented with FBS following overnight starvation. Data are presented as mean ± *s.e*., *n(cells)* indicates the number of independent cells measured. Masses are shown as absolute values. (b) Actomyosin cortex stiffness of PHOFs, CAHOFs, and MHOFs was probed using tipless AFM cantilevers to whose free ends 5 µm diameter silica beads had been glued. Cells were probed while adhering to fibronectin‐coated Petri dishes following a 5 and 120 min adhesion period in DMEM medium supplemented with FBS at 37°C. Extraction of the Young's Modulus is described in Methods. Data are presented as mean ± *s.d*., and *n* indicates the number of independent cells measured. (c) Mass and *Q_cell_* of wild‐type, pKO‐αV/β1 and pKO‐β1 fibroblasts adhering to fibronectin‐coated cantilevers. Before measurements, cells were incubated for 30 min with either 20 µm DMSO (control), 10 µm LY294002, or 100 nM Everolimus (Eve). Measurements were performed under cell culture conditions (37°C and 5% CO_2_) in DMEM medium supplemented with FBS following overnight starvation. Data are presented as mean ± *s.e*., and *n* indicates the number of independent cells measured. (d) Mass and *Q_spheroid_* of five single spheroids made of cancer‐associated fibroblast derived from the human rectum adhering to fibronectin‐coated cantilevers. Measurements were performed under cell culture conditions (37°C and 5% CO_2_) in DMEM medium supplemented with FBS. (e) Bright field images of measured spheroids adhering to the cantilever, shown right after attachment (0 min) and 180 min later. Scale bars, 100 µm. Statistical significance was evaluated using the Wilcoxon rank sum test. *P* values are given in figures, values >0.05 are considered non‐significant (n.s.).

In disease diagnosis and research, alterations in cell stiffness frequently signal pathological conditions [60, 61, 62]. For instance, cancer cells often reduce stiffness [63], while cells in fibrotic tissues stiffen [64]. Consequently, measuring the stiffness of entire cells, rather than only cellular compartments such as the cytoskeleton, could serve as an additional diagnostic indicator and contribute to a more comprehensive understanding of disease progression. To demonstrate the versatility and broader relevance of Q‐factor measurements for diagnosis, we applied our approach to characterize the growth and mechanical properties of primary fibroblasts in real‐time. We measured the mass and *Q_cell_* of single primary human ovarian fibroblasts (PHOFs, control), cancer‐associated human ovarian fibroblasts (CAHOFs), and metastatic human ovarian fibroblasts (MHOFs) over a 2 h period as they initiated and strengthened adhesion to fibronectin‐coated microcantilevers (Figure 5a). Compared to wild‐type mouse fibroblasts, which increased mass during this time course by ≈ 57% (Figure 2a and Figure S3), the mass of the ovarian fibroblasts remained largely unchanged across the three cell types during the measurement (Figure 5a). However, primary ovarian fibroblasts showed a considerably larger mass and considerably reduced *Q_cell_* values compared to wild‐type mouse fibroblasts. Amongst the ovarian fibroblasts, MHOFs exhibited a considerably lower *Q_cell_* compared to PHOFs and CAHOFs. Notably, this distinction could not be observed using conventional AFM indentation measurements (Figure 5b, Methods), which showed a lower Young's modulus for MHOFs compared to CAHOFs at 5 min and a higher Young's modulus at 120 min post‐attachment to the microcantilever.

In summary, these results show that cancer‐associated ovarian fibroblasts exhibit a considerably higher mass compared to non‐metastatic ovarian fibroblasts, while metastatic fibroblasts display softer mechanical properties than cancer‐associated and healthy fibroblasts. These drastic differences in mass and mechanical properties highlight the applicability of Q‐factor measurements to distinguish between metastatic and non‐metastatic cancer cells.

## 7 Fibroblasts Inhibited in Growth Lower the Q‐Factor

We next wanted to characterize whether cell growth regulation links to cell mechanical properties. We thus aimed to inhibit PI3K or mTOR, which take distinct roles in regulating cell growth, proliferation, and metabolism [65], and to measure cell mass and *Q_cell_*. To this end, we examined how PI3K/mTOR signaling modulates fibroblast growth by measuring the mass and *Q_cell_* of wild‐type fibroblasts, pKO‐αV/β1 fibroblasts, and pKO‐β1 fibroblasts attached to fibronectin‐coated microcantilevers for 2 h (Figure 5c). We chose to inhibit PI3K and mTOR because both kinases act in a common signaling cascade, yet they play distinct roles in mediating growth responses [66]. By comparing the effects of inhibiting PI3K and mTOR on different integrin knockout fibroblasts, we aimed to dissect the relative contributions of upstream vs. downstream signaling events of fibroblasts facilitating adhesion using different sets of integrins.

Before measurements, cells were pre‐incubated with DMSO (vehicle control), the PI3K inhibitor LY294002, or the mTOR inhibitor Everolimus. In wild‐type fibroblasts, both inhibitors markedly reduced cell mass over 2 h, from 2.82 ± 0.25 ng (mean ± s.e.) in the control to 1.82 ± 0.27 ng upon mTOR inhibition and to 1.43 ± 0.23 ng upon PI3K inhibition. Correspondingly, *Q_cell_* decreased from 1.78 ± 0.17 in the control to 1.49 ± 0.05 upon mTOR inhibition and 1.42 ± 0.07 upon PI3K inhibition. The mass of pKO‐αV/β1 fibroblasts reduced from 3.36 ± 0.34 ng in the control to 2.38 ± 0.34 ng upon mTOR inhibition and 2.65 ± 0.29 ng upon PI3K inhibition, while pKO‐β1 cells decreased mass from 3.25 ± 0.21 ng to 2.21 ± 0.26 ng upon PI3K inhibition but not upon mTOR inhibition. Notably, while pKO‐αV/β1 fibroblasts reduced *Q_cell_* values considerably, pKO‐β1 fibroblasts did not change their *Q_cell_* values in the presence of either inhibitor. The diminished drug effects in integrin knockout fibroblasts suggest that integrins are involved in regulating fibroblast growth through the PI3K/mTOR pathway. However, the measurements also show that integrins other than β1‐class integrins play roles in regulating cell mechanical properties, thus highlighting the need to further study the involvement of integrins in regulating cell growth and mechanical properties.

## 8 Monitoring Human Spheroids Reveals Stiffness Regulation During Growth

Next, we wanted to investigate whether our microcantilever approach can also be applied to monitor the growth and mechanical properties of larger cellular systems. To create spheroids, primary cancer‐associated fibroblasts (CAFs) were derived from the human rectum and seeded in microwell plates at a density of ≈ 100 cells per microwell two days prior to measurement. The CAF‐assembled spheroids were then attached to fibronectin‐coated cantilevers to monitor their growth and mechanical properties at cell culture conditions for 3 h (Figure 5d, left). During these measurements, the morphology of the single spheroids was optically recorded to follow their growth (Figure 5e). Directly after attachment, the individual spheroids showed a mass between 1000 and 2000 ng. This mass matched the expected mass of the spheroids at day 2 after seeding, given a median cell cycle length of 24 h for CAFs [67, 68], and an average single‐CAF mass of 4.65 ± 1.52 ng (Figure S7a). However, while two spheroids showed no growth, assuming they were in a resting period, three spheroids grew substantially almost doubling their mass within the recording time from which one maintained a steady‐state growth. The Q‐factor recorded for each spheroid *Q_spheroid_* did not change considerably and mainly fluctuated around mean values ranging from 2 to 3.2 (Figure 5d, right). Applying a low‐pass filter revealed cyclic variations of the Q‐factor, with a mean periodicity of 13.35 ± 5.45 min and an amplitude of Δ*Q*  =  0.36 ± 0.79. In comparison, the bare cantilever for these studies exhibited fluctuations of considerably smaller amplitudes of 0.027 ± 0.02 (Figure S7b,c). In contrast, wild‐type fibroblasts and pKO fibroblasts expressing distinct sets of integrins did not show such cyclic Q‐factor variations (Figure S8).

These findings suggest that the mass measured for individual spheroids is consistent with the predicted mass, thus proving the applicability of our experimental setup for the continuous monitoring of mass and growth of larger cellular systems. The cyclic fluctuations of the Q‐factor around mean values indicate that spheroids regulate their mechanical properties on relatively short timescales of ≈ 15 min, independently of whether they paused growth or grew. Thus, while regulating mass and growth, spheroids may regulate their stiffness to be kept constant. However, we note that these observations are based on a limited number of spheroids; establishing spheroid‐level regulation of mechanical properties as a general phenomenon will require larger cohorts, independent batches and, ideally, multiple donors.

## 9 Conclusions

We here introduced the experimental and theoretical framework to expand microcantilever‐based mass measurements of single cells and complex cellular systems and to simultaneously monitor their mass and Q‐factor at millisecond time resolution. The Q‐factor is widely used to measure the ratio of the energy dissipated by oscillating physical systems such as microcantilevers. It has been shown that such oscillating systems can be used to determine the Q‐factor of single bacterial cells [25] or of a population average in mammalian cells [29]. However, for a long time it remained unclear which cellular properties the Q‐factor describes. Here we show that the Q‐factor largely describes the mechanical properties of the cytoplasm, to which the actomyosin cortex contributes to a much lower – if not negligible – level. As such, the Q‐factor measurements provide a comprehensive measure of the mechanical properties of the entire cell or even of more complex cellular systems. Thereby we also show that the Q‐factor closely relates to the cytoplasmic Young's modulus of the cell. Advantageously, our approach allows monitoring the morphology, mass, and mechanical properties of cellular systems under culture conditions without the need for labeling or contrasting the sample and without side effects, such as bleaching, for the time course of multiple hours.

The Q‐factor is inversely related to the more conventional viscoelastic loss tangent tan *δ*. For a damped oscillator *Q_cell_* ≈ 1/tan *δ*  =  *G*′/*G*″, so that a high *Q_cell_* corresponds to a predominantly elastic, weakly dissipative response and a low *Q_cell_* to a more viscous one. The loss tangents, tan *δ*, which can be calculated from our optical‐tweezer and AFM microrheology data typically range from ≈ 0.1–0.4 (Figure S9), which lie in and below the range reported for the cytoplasm of mammalian cells and are consistent with the soft‐glassy, weak power‐law rheology of living cells [69]. For the cortex probed by AFM microrheology tan *δ* increases monotonically with frequency, and exceeds the 0.1–0.4 soft‐glassy band above ≈ 50 Hz. In contrast, the cytoplasm probed by optical tweezers shows tan *δ* to stay below the soft glassy range until 100 Hz. Because *Q_cell_* is an integrative, whole‐cell measure of mechanical energy dissipation, it is dominated by the cytoplasm as the largest and softest compartment but does not exclude contributions from other structures; in particular, perturbing the actin cytoskeleton can also alter nuclear prestress, positioning, and nucleus–cytoskeleton coupling [70]. We further note that the measurement itself is minimally invasive: the oscillation amplitudes are only a few Ångströms, and the photothermal actuation uses a low‐power (10 µW) laser, and mammalian cells remain healthy and continue to divide on the cantilever for days [27, 37], so that we detect no signs of measurement‐induced perturbation under these gentle conditions.

The nucleus deserves particular consideration here. As one of the mechanically most prominent organelles, it is coupled to the actomyosin cytoskeleton and, through it to cell–matrix adhesions by the LINC (linker of nucleoskeleton and cytoskeleton) complex, which links the nuclear lamina to actin, microtubules and intermediate filaments via SUN and nesprin proteins [71, 72]. Disrupting the actin cytoskeleton therefore does not act on the cytoplasm alone, but can also alter nuclear prestress, positioning, and nucleus–cytoskeleton coupling, further modulated by cell‐cycle stage, chromatin condensation, lamin A/C organization, and nuclear shape. Part of the *Q_cell_* changes measured upon cytoskeletal perturbation could thus arise from altered nuclear mechanics rather than cytoplasmic rheology alone. While the cytoplasm, as the largest and softest compartment, is expected to dominate the dissipation captured by *Q_cell_*, we do not exclude a nuclear contribution. Disentangling this contribution is an important next step that our label‐free, real‐time approach is well suited to pursue, e.g., through LINC‐complex disruption, lamin perturbation, or correlation of *Q_cell_* with cell‐cycle stage.

Interestingly, we find that mammalian cells and cellular systems maintain a relatively constant Q‐factor during growth. This consistency likely indicates that single cells and cellular systems regulate their mechanical properties to avoid large swings. We further observe that spheroids oscillate the Q‐factor around mean values, thus suggesting them to actively regulate their mechanical properties on short timescales. Prompted by this observation, we reanalyzed our single cell measurements for which we could detect no Q‐factor fluctuations (Figure S8). Although the detection of Q‐factor fluctuations of single cells may be limited by the sensitivity of our experimental setup, this unique observation suggests that cells can collectively regulate the mechanical properties of more complex cellular systems. One upcoming challenge may thus be to explore in real‐time how complex cellular systems adjust mechanical properties in response to their development, state, or various internal and external stimuli and perturbations. Our nanotechnological approach thus opens new avenues for deeper understanding of cellular mechanics and their regulation, with significant implications for biophysics, cell biology, physiology, and medicine.

## 10 Experimental Section/Methods

### 10.1 Cell Culture

Wild‐type, pKO‐αV/β1, pKO‐αV, and pKO‐β1 mouse fibroblasts [49] were kindly provided by R. Fässler. HeLa cells (Kyoto) were kindly provided by A. A. Hyman. Human primary ovarian fibroblasts (#H‐6072), human ovarian tumor‐associated fibroblasts (#HC‐6072), and human metastatic ovarian tumor‐associated fibroblasts (#HC‐6265) were purchased in frozen vials from Cell Biologics, US. Mouse and human fibroblasts were grown on T25 tissue culture flasks (#TCF012050, JetBiofil, China) and cultured in Dulbecco's Modified Eagle Medium (DMEM) medium containing 4.5 g l^−1^ glucose, L‐glutamine and sodium pyruvate (#10‐013‐CV, Corning, US) and supplemented with 10% (v/v) fetal bovine serum (FBS, #97068‐085, Avantor, US), 100 U mL^−1^ penicillin and 100 µg mL^−1^ streptomycin (#30‐002‐CI, Corning, US). pKO‐αV/β1, pKO‐αV, and pKO‐β1 cells were cultured on tissue culture flasks previously incubated overnight with a solution of 50 µg mL^−1^ bovine plasma fibronectin (#341631‐5MG, Merck, Germany). All cells are maintained under 5% CO_2_ at 37°C in a humidified incubator and passaged at 80%–90% confluency by washing 1x with warm PBS (#21‐040‐CV, Corning, US) and detached from tissue culture flasks with 0.25% (w/v) trypsin‐EDTA (#25200056, Gibco, US). Human cancer rectal tissue was obtained from patients undergoing surgery. A written informed consent was obtained from all patients. The study was performed in accordance with the Helsinki declaration and approved by the ethics committee at the Faculty of Medicine at Ludwig‐Maximilian‐University (no. 025‐12) and the Bavarian state medical association (no. 11142) within the framework of the non‐profit foundation HTCR (Munich). Tumor tissue was dissociated using the tumor dissociation kit (#130‐095‐929, Miltenyi, Switzerland) and seeded in mesenchymal medium (#C‐28009, PromoCell, Germany) in a 6‐well plate. After one week, Cancer‐associated fibroblasts (CAFs) appeared attached and were subsequently passaged. To generate 3D spheroids, Gri3D 96‐well plates (SUN bioscience, Switzerland), each containing 71 microwells of 400 µm in diameter per well, were used. Hydrogel arrays were equilibrated with 100 µl of mesenchymal medium for at least 30 min at room temperature and then removed. 2D CAFs were dissociated using TrypLE (#12563029, Thermofisher, US) and centrifuged at 400 × g for 4 min, and counted. Cells were seeded at a density of 100 cells per microwell in 100 µL of mesenchymal medium per well. Plates were incubated at 37°C in 5% CO_2_ for 20 min, followed by the addition of 100 µL of medium. Plates were maintained at 37°C for further use.

### 10.2 Microcantilever Preparation for Resonance Frequency and Q Factor Measurements

For single cell measurements, tipless silicon microcantilevers (HQ:NSC35/tipless/No Al, Mikromasch, Germany) were cut using a focused ion beam (FIB, Helios NanoLab 650, FEI Company, US). FIB‐modified cantilevers present an outer rectangular geometry of 120 × 45 × 2 µm^3^ with a rectangular window of ≈ 80 × 30 × 2 µm^3^ being cut in the middle of the cantilever beam as described [28]. For spheroid measurements, 500 × 100 × 1 µm^3^ tipless silicon microcantilevers (#Arrow‐TL1‐50, NanoAndMore, US) were FIB‐cut to a rectangular shape. Cantilevers were calibrated in air using the Sader method [73]. Before use, cantilevers were cleaned in 95% sulfuric acid for 15 min at room temperature (21°C), then rinsed in ultrapure water and dried with precision wipes (#34120, Kimberly‐Clark, US). Then, cantilevers were plasma treated (PDC‐32G, Harrick Plasma, US) for ≈ 15 min and subsequently incubated overnight at 4°C in PBS with the substrate of interest: 2 mg mL^−1^ ConA (#C2010‐25MG, Sigma, US) or 50 µg mL^−1^ fibronectin.

To characterize the dependence of the Q‐factor shift on resonance frequency *f_N_* (Figure 1d,e), cantilevers of the same material, thickness, and width but of different lengths were FIB‐milled to span nominal resonance frequencies of ∼5, 15, 45, 80, and 100 kHz. For all subsequent single‐cell measurements, cantilevers with a resonance frequency of ∼45 kHz were used, as they provide the best compromise between Q‐factor and mass sensitivity.

### 10.3 Picobalance Setup

Single‐cell resonance frequency and Q‐factor measurements were conducted using the picobalance setup as described [27, 28]. In brief, microcantilevers were mounted onto a customized AFM head, which was equipped with piezo motors for movement along the *z*‐axis. Additionally, the AFM head features a low‐power intensity‐modulated blue laser (405 nm) for the photo‐actuation of the microcantilever. The blue laser power was set to 10 µW, with its temperature stabilized at 25°C by a laser diode controller. The deflection of the free end of the microcantilever was detected by reflecting an infrared laser (852 nm, ≈ 165 µW) onto a four‐quadrant position‐sensitive photodiode (S5980 Si PIN, Hamamatsu, Japan). The picobalance was mounted on an inverted optical microscope (Zeiss Observer Z1), featuring a digital camera (Hamamatsu Orca Flash 4.0) and a 10x/0.3 M27 Plan‐Apochromat objective (Zeiss). This configuration allowed for the acquisition of both differential interference contrast (DIC) and fluorescent images during cell mass measurements. The picobalance was equipped with an environmental chamber to maintain cell culture conditions (37°C and 5% CO_2_) while preventing medium evaporation during the cell mass measurement.

### 10.4 Resonance Frequency, Total Mass, and Q‐Factor Measurements

For short term (5 min) measurements with compounds (Figure 2), cells were pre‐incubated for 30 min with 5 µm latrunculin A (LatA, #L12370, Thermofisher, US), 5 µm cytochalasin D (CytoD, #PHZ1063, Thermofisher, US), 5 µm jasplakinolide (Jasp, #J7473, Thermofisher, US), or 20 µm IPA3 (#I2285‐5MG, Merck, Germany). For time‐dependent mass and Q‐factor measurements (Figure 3), cells were subjected to serum starvation overnight the day prior to experiments in DMEM GlutaMAX (#10569010, Thermofisher, US), which included 100 U mL^−1^ penicillin and 100 µg mL^−1^ streptomycin. This procedure aimed to minimize cell cycle variations during the measurements [74, 75]. On the day of the measurement, a glass‐bottomed Petri dish (Ibidi, µ‐Dish 35 mm, low) was affixed to the picobalance stage and filled with 1.5 mL phenol red‐free high‐glucose DMEM medium (#31053028, Thermofisher, US) warmed up at 37°C. This medium was supplemented with 1 mm sodium pyruvate, 4 mm GlutaMAX (#35050061, Thermofisher, US), 100 units mL^−1^ penicillin, 100 µg mL^−1^ streptomycin, and 10% (v/v) FBS. A coated cantilever (ConA for short term and fibronectin for long term measurements) was then mounted on the cantilever holder and placed in the Petri dish, which contained the medium at 37°C. Following this, the actuation blue and readout infrared lasers were precisely positioned on the cantilever, and the system was equilibrated for 30 min. During this time, serum‐starved cells were washed in PBS, detached using 0.25% (w/v) trypsin‐EDTA, and allowed to recover for 15 min in the same medium that had been previously added to the Petri dish. Subsequently, the suspended cells were introduced into the Petri dish positioned on the picobalance. Mass measurements were conducted using a continuous sweep mode, as detailed [76]. Initially, a frequency sweep was executed to identify the resonance frequency *f_N_* of the bare cantilever without cells attached. This sweep acquisition was controlled by customized LabVIEW software, where each sweep was centred around the *f_N_* the cantilever and acquired over a frequency range of ± 19.5 kHz. Each sweep consisted of 256 data points, with a data point acquired every 10 ms. Thereafter, the cantilever was brought into contact with a target cell or spheroid for 5–10 s. For single‐cell measurements, typically only cells with round morphologies and diameters between ≈ 13 and 18 µm were chosen for measurements. Subsequently, the cantilever was retracted by 200 µm to completely detach the cell or spheroid attached to the cantilever from the glass bottomed Petri dish [77] and to prevent hydrodynamic effects on the cantilever's movement [78]. Another frequency sweep was then recorded to determine *f_N_* of the cantilever with the cell or spheroid attached. Subsequently, frequency sweeps were automatically recorded at intervals of ≈ 10 s for a predetermined duration. Concurrently, DIC microscopy images of the cell or spheroid on the cantilever were captured every 5 min. These images collected during the long‐term mass measurements were used to estimate the position and spreading area of the cell on the cantilever using an in‐house software [76].

To extract the inertial mass and the Q‐factor of the attached cell or multicellular system, the amplitude curves collected during the frequency sweeps were fit using the formula below to extract *f_N_* and Q‐factor *Q_tot_* of microcantilever and attached cell or multicellular system [24, 27]:

(1) $$A\left(f\right)=\frac{a}{\sqrt{{\left(f_{N}^{2}-f^{2}\right)}^{2}+{\left(\frac{f_{N}\cdot f}{Q}\right)}^{2}}}+a_{1}\cdot f+a_{2}$$

where *A* is the amplitude, *f* the excitation frequency, *Q* the Q‐factor of cantilever *Q_cant_* or *Q_tot_*, *a* a parameter relating to the amplitude of the excitation signal, and *a*_1_ and *a*_2_ representing fitting parameters, which take external vibrations into account. Afterwards, the resonance frequency shift measured upon cell attachment Δ*f_N_* was converted into the mass Δ*m_cell_* of the cell attached to the microcantilever using the following formula [33]:

(2) $$\Delta m_{cell}=\frac{k}{4\pi^{2}}\cdot \left(\frac{1}{{\left(f_{N}\;-\;\Delta f_{N}\right)}^{2}}\;-\;\frac{1}{f_{N}^{2}}\right)$$

*Q_tot_* is then determined by contributions from both the cantilever *Q_cant_* and the attached cell *Q_cell_* as the following [79]:

(3) $$\frac{1}{Q_{tot}}=\frac{1}{Q_{cant}}+\frac{1}{Q_{cell}}$$

We decompose the effect of the cell on the Q‐factor into two components: the mass contribution and the finite body contribution. The term “cell mass” refers to the inertial mass that the cell adds to the cantilever. Thereby the cell mass is considered as a point mass located at a specific position on the cantilever. However, an animal cell is not a point mass and comes along with its own shape, size, and heterogeneous properties (e.g., stiffness, damping). Therefore, we introduce the term “cell body” to capture these effects.

To derive the theoretical Q‐factor *Q*_*tot*,*theory*_ upon attaching a point mass to the microcantilever we consider the ratio Δ_*mass*_ of cell mass *m_cell_* and cantilever mass *m_cant_* (without cell) [34]:

(4) $$\Delta_{mass}=\frac{m_{cell}}{m_{cant}}$$

*Q*_*tot*,*theory*_ is then given by:

(5) $$Q_{tot,theory}=\sqrt{Q_{cant}^{2}\cdot \left(1+\Delta_{mass}\right)+\frac{1}{\Delta_{mass}}}$$

This is a simplified model where the cell mass influences *f_N_* of the cantilever solely by adding weight. Under the assumption that the cell is a point mass, the shift in Q‐factor can be described by:

(6) $$\frac{1}{Q_{tot,theory}}=\frac{1}{Q_{cant}}-\frac{1}{Q_{mass}}$$

where *Q_cant_* is the Q‐factor of the bare cantilever without cell, and *Q_mass_* represents the Q‐factor contribution of *m_cell_* to the theoretical framework where the *m_cell_* is considered as a point mass. When both the finite cell body and mass contribute to the total Q‐factor *Q_tot_*, then *Q_tot_* can be expressed as:

(7) $$\frac{1}{Q_{tot}}=\frac{1}{Q_{cant}}+\frac{1}{Q_{cell}}-\frac{1}{Q_{mass}}$$

From this, the Q‐factor contribution of the cell body can be isolated:

(8) $$\frac{1}{Q_{cell}}=\frac{1}{Q_{tot}}-\frac{1}{Q_{cant}}+\frac{1}{Q_{mass}}$$

To estimate the Young's modulus of the cell *E_cell_*, the following relationship derived from a harmonic oscillator model involving the Q‐factor of the cell body was used:

(9) $$k_{cell}=\frac{{Q_{cell}}^{2}\cdot c^{2}}{m_{cell}}$$

Here, *k_cell_* is the effective spring constant of the cell and *c* a damping constant related to damping of the overall system (cantilever + cell) in fluid, which is extracted by finite element simulations.

Thus, *E_cell_* can be approximated as:

(10) $$E_{cell}=\frac{k_{cell}\cdot d_{cell}}{A_{cell}}$$

where *d_cell_* is the diameter and *A_cell_* the spreading area of the cell attached to the cantilever determined via optical microscopy.

The normalized Q‐factor shift reported in Figure 2c is defined as the Q‐factor change Δ*Q_tot_* upon attachment of a bead or cell to the microcantilever divided by the Q‐factor of the bare cantilever measured immediately before attachment. This normalization removes the cantilever‐to‐cantilever variation in the absolute Q‐factor of the FIB‐sculpted cantilevers and renders the shifts measured with different cantilevers directly comparable.

### 10.5 AFM‐Based Indentation Experiments

Silica microbeads with a diameter of 5 µm (PSI‐5.0, Kisker Biotech, Germany) were glued with UV glue (OP‐29, Dymax, US) to the free end of V‐shaped NPO‐D cantilevers having nominal spring constants of 0.06 N m^−1^ (NP‐010, Bruker, Germany) and cured under UV light for 30 min. Single cantilevers were plasma treated for 2 min using a plasma cleaner (PDC‐32G, Harrick Plasma, US) before each experiment. An AFM (CellHesion 200, JPK Instruments, Germany) mounted on an inverted optical microscope (AxioObserver Z1, Zeiss, Germany) was used. To extract the spring constant, cantilevers were calibrated using the thermal noise method [80] implemented in the AFM software (JPK SPM V5, JPK Instruments, Germany). The setup was placed in a heat chamber (The Cube, Life Imaging Services, Switzerland) to maintain a temperature of 37°C. Fibroblasts were washed with PBS and detached by incubation with 0.25% (w/v) trypsin‐EDTA. Later, fibroblasts were resuspended in serum‐free DMEM medium supplemented with 20 mm HEPES (Thermofisher, US). To evaluate the change in elastic modulus upon adhesion, suspended fibroblasts were plated on a fibronectin‐coated dish, which had been mounted on the AFM setup, and let adhere for 5 min. Single fibroblasts were indented by pushing microbeads on top of their nucleus until reaching a setpoint of 8 nN. The Petri dish was then incubated at 37°C and 5% CO_2_. After 2 h, the same dish was placed under the AFM setup and single fibroblasts were indented again on top of their nucleus until reaching a setpoint of 8 nN. Force‐distance curves were corrected for cantilever deflection. To extract the elastic modulus, the first 800 nm of the indentation depth δ preceding the contact point between cell and microbead were fitted with the Hertz contact model for spherical indenters [7]:

(11) $$F\left(\delta \right)=\frac{4\sqrt{R}}{3}\;\;\cdot \frac{E}{\left(1-\nu^{2}\right)}\cdot \delta^{\frac{3}{2}}$$

where *F* is the force, *R* is the radius of the bead, ν is the Poisson's ratio (assumed 0.35 for cells according to literature [81, 82]) and *E* the Young's modulus of cells.

### 10.6 AFM‐Based Parallel Plate Assays

Stiffness measurements were performed using an AFM (Nanowizard II equipped with a CellHesion module from JPK Instruments, Berlin), mounted on an inverted microscope (Observer.Z, Zeiss). The AFM setup included a Petri dish‐heater to maintain the cell culture medium at 37°C. Parallel plate measurements were conducted of rounded cells sandwiched between the Petri dish and a 225 µm long FIB fabricated wedged cantilever [8]. Prior to the experiments, glass‐bottom Petri dishes were washed with ethanol and deionized water, then coated with 50 µg mL^−1^ fibronectin in PBS overnight at 4°C. The cantilever was plasma cleaned (PDC‐32G, Harrick Plasma, US) and incubated overnight at 4°C with 50 µg mL^−1^ of a fibronectin fragment lacking the essential integrin‐binding site (FNIII7‐10ΔRGD) [51] in PBS. The spring constant of the cantilever was determined using the thermal noise method prior to the experiments [73]. Fibroblasts were detached using 0.25% (w/v) trypsin‐EDTA, pelleted, and resuspended in DMEM supplemented with 20 mM HEPES. Suspended fibroblasts were allowed to recover from trypsin‐EDTA treatment for at least 30 min [83]. Subsequently, they were pipetted onto the fibronectin‐coated Petri dish and allowed to settle. The wedged cantilever was positioned above a rounded fibroblast and approached the fibroblast at a speed of 5 µm s^−1^ until detecting a force of 25 nN. The cantilever was then retracted. During approach and retraction, a force‐distance curve was recorded. Afterward, the cantilever was positioned above another rounded fibroblast to repeat the measurement. The apparent contact stiffness of each cell was quantified as the slope of a linear function fitted to the approach force‐distance curve between 5 and 20 nN.

### 10.7 AFM‐Based Microrheology Experiments

Suspended cells were plated on fibronectin‐coated glass‐bottom Petri dishes (FluoroDish, WPI, US) and were placed into an incubator for 2 h at 37°C and 5% CO_2_ to let the cells adhere. The Petri dish with adhered cells was then mounted on the combined AFM (JPK NanoWizard V, Bruker, Germany) and inverted microscope (AxioObserver Z1, Zeiss, Germany) setup. To maintain the cells at culture conditions (37°C and 5%CO_2_) during the experiment, the setup was kept in a temperature‐controlled chamber (The Cube, Life Imaging Services, Switzerland), and the dish with 2 mL of DMEM medium was perfused with a humidified gas mixture comprising synthetic air with 5% CO_2_ [37]. A bead with a diameter of 5 µm (PSI‐5.0, Kisker Biotech, Germany) was glued to the free end of a triangular cantilever with nominal spring constant 0.06 N m^−1^ (NP‐010, Bruker, Germany). To measure the rheological properties of the cells, the bead was indented at 1 µm s^−1^ on top of the nucleus of single cells until reaching a setpoint of 2.5 nN and oscillated at frequencies of 1, 2, 5, 10, 20, 50, 100, 200, and 500 Hz with an amplitude of 50 nm for 25 periods. The rheological data were analyzed with Bruker software (DP Version 8.0.157). To calculate the complex storage and loss modulus, the software follows the method implemented by Mahaffy et al. [84].

### 10.8 Imaging of Single Cell Volume, Actomyosin Cortex and Intermediate Filaments

8‐well slides (#80827‐IBI, Ibidi, Germany) were plasma cleaned for 10 min and coated with 200 µL of 50 µg mL^−1^ fibronectin in PBS solution at 4°C overnight. The following day, cells were seeded on the coated slides at a density of 5′000 cells/well. 15, 30, 60, and 120 min post seeding, the cells were fixed using a 4% (w/v) paraformaldehyde in PBS solution for 10 min at 37°C. After fixation, cells were washed with excess PBS and permeabilized using 5% (w/v) BSA/0.2% (v/v) Triton X‐100/1X PBS for 1 h at room temperature. After permeabilization, cells were washed with excess PBS and incubated with a solution of 5 µm CellTracker Green CMFDA for total volume imaging (Figure S3) and a solution of primary antibodies diluted in 1% (w/v) BSA/0.2% (v/v) Triton X‐100/1X PBS at 4°C overnight for actomyosin cortex imaging (Figure S4). Primary antibody dilutions were 1/400 Alexa Fluor Plus 647 phalloidin (#A30107, Invitrogen, US), 1/200 phospho‐MYL9 (Ser19) (#MA5‐15163, Invitrogen, US), and 1/400 anti‐vimentin (#ab24525, Abcam, UK). The following day, cells were rinsed 3 times with the 0.2% (v/v) Triton X‐100/1X PBS solution and incubated with the secondary antibodies diluted in 1% (w/v) BSA/0.2% (v/v) Triton X*‐100/1X *PBS for 1 h at room temperature in a dark environment. The secondary antibodies used were Goat Anti‐Mouse IgG H&L Alexa Fluor 488 (#ab150113, Abcam, UK), Goat Anti‐Chicken IgY H&L Alexa Fluor 568 (#ab175477, Abcam, UK), Goat Anti‐Rabbit IgG Fc Alexa Fluor 750 (#ab175735, Abcam, UK) were used at a 1/500 dilution and DAPI (#62248, Thermofisher, US) at 1/1′000 dilution. Afterwards, the samples were washed in PBS and imaged with a Zeiss LSM980 point scanning confocal microscope (Zeiss, Germany) using a Plan‐Apochromat 63X/1.4 Oil DIC M27 objective (#420782‐9900‐000, Zeiss, Germany). Signals were collected sequentially in Airyscan super‐resolution mode. Images were processed using the ZEN Blue 3.8software (Zeiss) and 3D Airyscan processing with default settings.

### 10.9 Optical Tweezer‐Based Microrheology

Optical tweezer experiments were conducted on a commercial C‐Trap optical tweezers (Lumicks, Netherlands). Following the same protocol as for the magnetic tweezer experiments, cells were seeded at ≈ 50% confluence on glass bottom Petri dishes with a diameter of 60 mm in DMEM culture medium and incubated overnight. The following day, 0.49 µm carboxyl latex beads (#C37269, Thermo Fisher Scientific, US) were added to the Petri dish at a concentration of 1 µg mL^−1^ and incubated again overnight to allow for the beads to be endocytosed by the cells. The following day, cells were first washed in warm PBS to remove the excess of beads and 1 mL of DMEM imaging medium (#21063‐029, Gibco‐Life technologies, US) warmed up at 37°C was subsequently added to the Petri dish for measurements. Importantly for these measurements, the imaging medium needs to cover only the glass bottom of the Petri dish without overflowing the plastic sides. In addition, a 1 mm thin glass cover slip (#16004‐422, VWR International, Germany) was added on top of the Petri dish with medium, to separate the condenser of the C‐Trap setup from the cell culture medium. The Petri dish was then aligned perpendicular in the C‐Trap setup between the water objective (60x, NA 1.2, MRD07602, Nikon, Japan) coming from the bottom and the matching condenser (60x, NA 1.4, used with Type A immersion oil, Leica) coming from the top. The Petri dish was positioned in between the two such that the condenser can adequately collect photons from the infrared (IR) trapping beams (1′064 nm) and project on to position‐sensitive detectors for accurate trap force measurement. The trap force and position data were acquired at a rate of 78’125 Hz. Both the objective and the condenser were pre‐warmed to 30°C–35°C from the temperature control unit for 30 min prior to experiments. The IR trapping power was typically set at 100% trapping laser and 15% of the 20 W overall power, leading to ≈ 3 W. These settings correspond to a trap stiffness of ≈ 0.02 pN nm^−1^. For these experiments, the power was split between trap 1 (T1) and trap 2 (T2) in a 100‐0 ratio, as only T1 was utilized. The bright field LED intensity was adjusted between 80% and 100% as needed. During each measurement, a trapped bead was oscillated for 7 s at 1 Hz, for five seconds at 2, 5, 10 Hz and for 3 s at 20, 50, 100 Hz at an amplitude of 500 nm. The python code used is published on GitHub by Lumicks [https://github.com/lumicks/bluelake‐scripting/blob/master/scripts/workflow/mcl_oscillation.py]. During the oscillations, the trap force, trap position, and bead position were tracked in both x and y directions. To extract the *G*′ and *G*′′ modulus from the data recorded, we first calculated the force *F*, trap displacement, and bead displacement *x* signals by computing the point‐by‐point Euclidean distance of each x and y point. Then, the phase shift Δϕ is calculated between the total force signal and trap position sinusoidal signals. The viscoelastic moduli were calculated as $G^{\prime }\left(\omega \right)=\left(\frac{F_{max}}{6\pi rx_{max}}\right)\cos\left(\Delta \phi \right)$ and $G^{\prime \prime }\left(\omega \right)=\left(\frac{F_{max}}{6\pi rx_{max}}\right)\sin\left(\Delta \phi \right)$ [59], where *F_max_* is the maximum amplitude of the force signal *F* and *x_max_* is the maximum amplitude of the bead displacement signal at each oscillation. When dealing with viscoelastic materials, the storage and loss moduli *G*′and *G*′′ represent the real and imaginary parts of the complex shear modulus G*, which is:

(12)

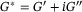




To estimate the Young's modulus of the cell cytoplasm from optical tweezers data, under the assumption that the cytoplasm behaves as a uniform material, the relationship between the complex shear modulus G* and Young's modulus *E* can be expressed as:

(13) $$E=2G^{*}\left(1+\nu \right)$$

where ν is the Poisson's ratio and is chosen to be 0.35 for mammalian cells [81, 82].

### 10.10 COMSOL Cantilever–Cell System Simulations

Finite element analysis (FEA) of a cell attached to the cantilever was performed using the solid mechanics module of COMSOL Multiphysics 5.5. The cantilevers were modelled as silicon (Young's modulus = 170 GPa), while the cell was modelled as a custom material with a 200 nm thick shell of varying Young's moduli and density. The cantilever together with the cell attached were surrounded by a 200 × 200 × 200 µm^3^ water cube, which is set to behave according to a linearized Navier‐Stokes model [85]. In the FEA setup, the cantilever motion was simulated with one end fixed as a constraint and a 1 nN boundary load applied in z‐direction to the opposite end [28]. Additionally, an aeroacoustic‐structure boundary was defined at the interface between the cantilever and the surrounding water within the COMSOL Multiphysics module. Frequency sweeps of the cantilever were acquired by performing a frequency domain study between 20 and 80 kHz in steps of 0.5 kHz.

### 10.11 Statistical Analysis

Data were processed as described in the corresponding Methods sections. Only mitotic cells were excluded from analysis; no additional observations were removed as outliers. Data are presented as mean ± standard deviation (s.d.) or mean ± standard error of the mean (s.e.m.), as indicated in the figure legends. Sample sizes are reported in the figures, with *n* representing independent cells, beads, or spheroids. Optical‐tweezer cycles were summarized at the bead level, and spheroid measurements were analyzed descriptively because of the limited sample size.

Statistical significance was assessed using two‐sided Wilcoxon rank‐sum tests for independent groups and Wilcoxon signed‐rank tests for paired measurements. Where multiple comparisons were performed against a common reference, *p* values were adjusted using the Holm method. Differences were considered not significant for *p* > 0.05 and significant at ^*^ *P* ≤ 0.05, ^**^ *P* ≤ 0.01, and ^***^ *P* ≤ 0.001. Analyses were performed using MATLAB R2022b.

### 10.12 Data Analysis

For data analysis, only mitotic cells were discarded while all other cell measurements were included. The mass and spreading area of single cells were calculated using the pyIMD software (version 0.1.3) [76]. Wilcoxon rank‐sum test was used to determine significant differences between mean values. Differences were considered not significant (n.s.) if *p* > 0.05 and significant if ^*^ *P* ≤ 0.05, ^**^ *P* ≤ 0.01, ^***^ *P* ≤ 0.001. All statistical analyses were performed using MATLAB R2022b.

### 10.13 Transparency Statement

Generative AI tools were not used to generate any scientific content, data, analyses, figures, or conclusions of this work. AI‐assisted and standard language‐editing tools were used only to polish the grammar and readability of author‐written text; all edits were reviewed and approved by the authors, who take full responsibility for the manuscript.

## Author Contributions

I.I. and D.J.M. designed the study and discussed the experimental setup. I.I. conducted and analyzed the picobalance and optical microscopy experiments with the help of S.H. I.I., G.A. and N.S. performed and analysed AFM experiments. I.I., T.K. and D.A.W. designed and carried out the optical tweezers experiments. M.C.R. and M.P.L. prepared spheroids and contributed to the spheroid data acquisition. I.I. and D.J.M. wrote the first draft of the manuscript. All authors reviewed and approved the manuscript.

## Funding

NCCR Molecular Systems Engineering of the Swiss National Science Foundation (SNFS, Grant 51NF40‐205608), Swiss National Science Foundation (Grant 31003A_182587, 310030_215690), Swiss Innovation Agency (InnoSuisse Grant 56599.1 IP‐LS)

## Conflicts of Interest

D.J.M. has filed two patents related to cell picobalance technology and its applications (US20170052211A1 and WO/2015/120,991). M.C.R. and M.P.L. are employees of Hoffmann‐La Roche. The remaining authors declare no conflict of interest.

## Supporting information




**Supporting File**: advs77926‐sup‐0001‐SuppMat.pdf.

**Supporting File**: advs77926‐sup‐0001‐SuppMat.xlsx.

## Data Availability

The raw data that support the findings of this study are available from the public repository of the ETH Zurich Research Collection at https://doi.org/10.3929/ethz‐c‐000804063. The source data is available as Supplementary Information. All other information is available upon reasonable request from the corresponding author of this study.
