# Supplementary material for: Real‐Time Monitoring of Mass and Mechanical Properties in Single Cells and Multicellular Spheroids
Source: Adv Sci (Weinh). 2026 Sep 27:e77926. Online ahead of print. doi: 10.1002/advs.77926 (PMC13616342; doi:10.1002/advs.77926)
Supplement: Supplementary file 1 [file ADVS-9999-e77926-s002.pdf]

Supporting Information

Real-Time Monitoring of Mass and Mechanical Properties in Single Cells and Multicellular Spheroids

*Ilaria Incaviglia, Giulia E. M. Ammirati, Sophie Herzog, Tommy Krug, Nico Strohmeyer, Miguel Camacho Rufino, Matthias P. Lutolf, David A. Weitz, and Daniel J. Müller**

a

$f_{N,cant}$	5 kHz		15 kHz		45 kHz		80 kHz		100 kHz	
	w/o cell	w/ cell	w/o cell	w/ cell	w/o cell	w/ cell	w/o cell	w/ cell	w/o cell	w/ cell
Q_{tot} mean	2.24	2.19	2.93	2.77	4.36	3.65	6.31	5.67	5.83	4.79
Q_{tot} ± *s.d.*	0.30	0.28	0.29	0.28	0.16	0.28	0.44	0.44	0.31	0.40

$f_{N,cant}$	5 kHz	15 kHz	45 kHz	80 kHz	100 kHz
Q_{tot} shift mean	0.05	0.16	0.71	0.64	1.04
Q_{tot} shift ± *s.d.*	0.04	0.09	0.25	0.34	0.29

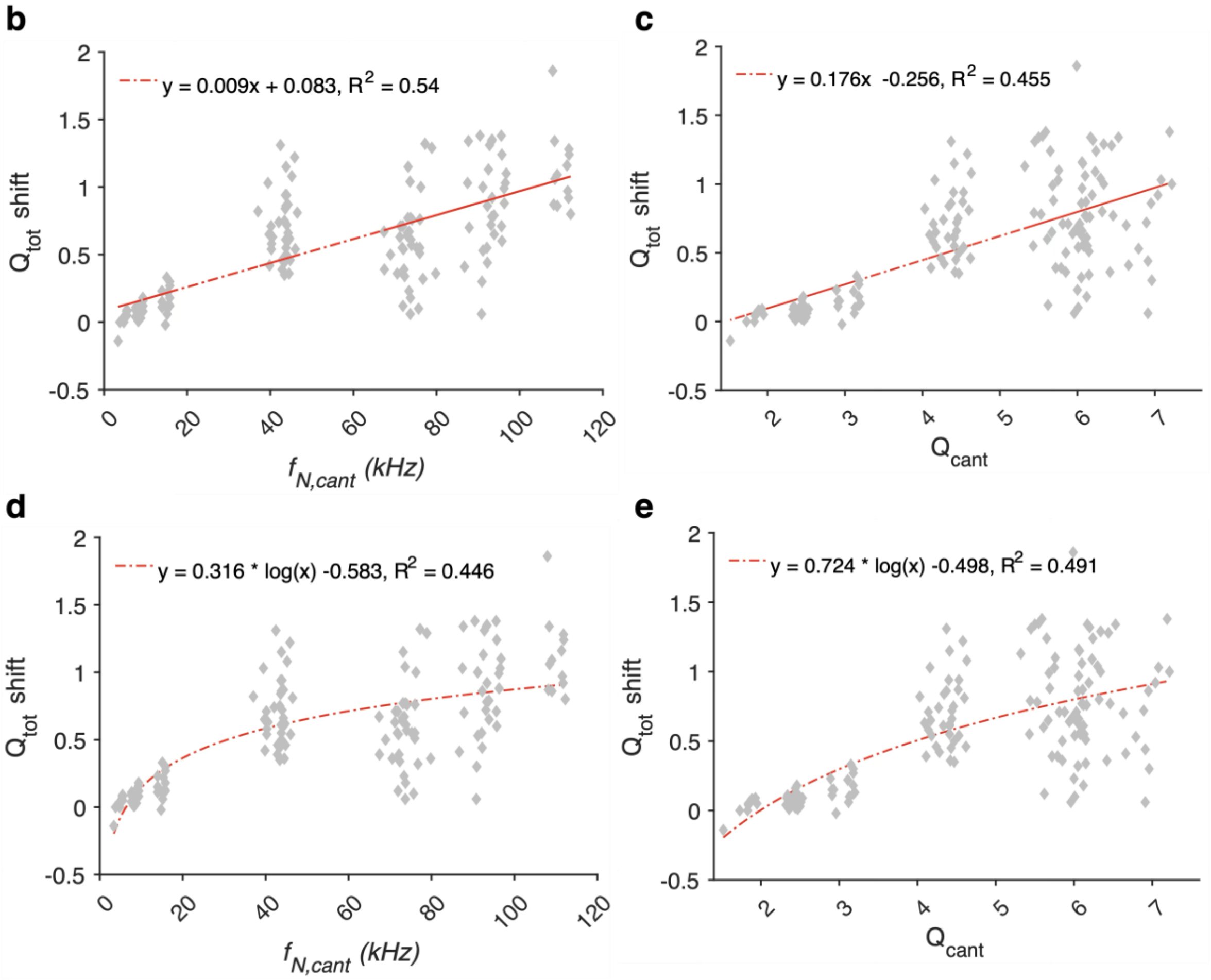

Supplementary Figure S1. Q-factor shift induced by the attachment of single HeLa cells to the microcantilevers. a, Table reporting mean and *s.d.* of data shown in Figure 1d,e. **b-e,** Q_{tot} shifts of ConA-coated cantilevers measured upon HeLa cell attachment were fit with a linear and logarithmic fitting and plotted against the initial cantilever resonance frequency $f_{N,cant}$ and the initial Q_{cant} of the cantilever prior to cell attachment. Measurements were performed under cell culture conditions (37°C and 5% CO_2) in DMEM medium supplemented with FBS following overnight starvation of the cells. Data are presented as the mean ± *s.d.*, and *n* indicates the number of independent cells measured. A maximum of 3 cells were measured for each cantilever used. The data reveal that Q_{tot} exhibits a stronger linear correlation with the initial cantilever resonance frequency, whereas its dependence on the initial Q_{cant} of the cantilever is better described by a logarithmic relationship.

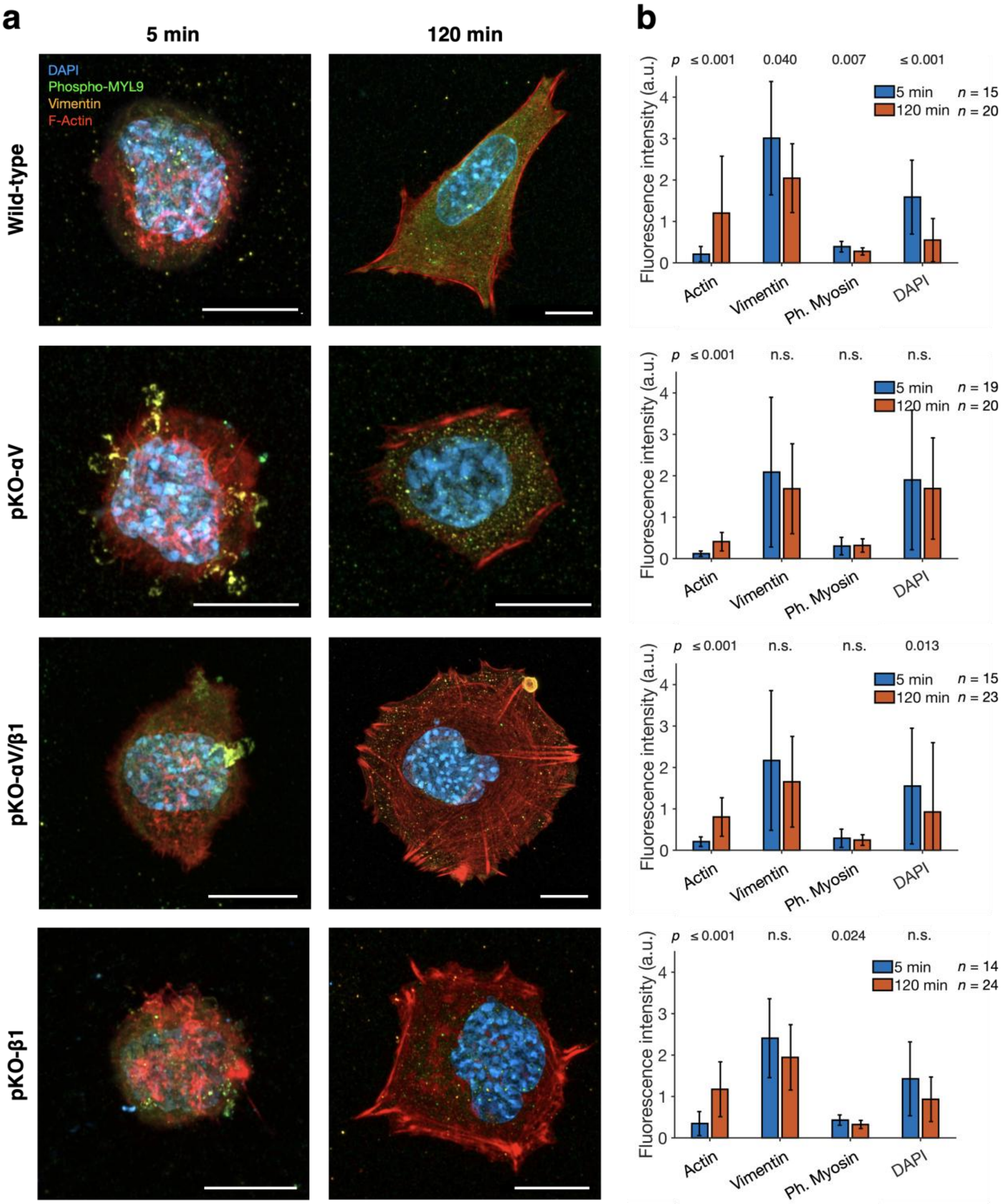

Supplementary Figure S2. Dynamics of cytoskeletal components of fibroblasts spreading on fibronectin. a, Representative fluorescence images of wild-type, pKO-αV, pKO-αV/β1, and pKO-β1 fibroblasts spreading on fibronectin-coated 8-well slides, captured at 5 and 120 min post-seeding. Fluorescent markers show F-actin (red), phospho-myosin (green), vimentin (yellow), and nuclei (DAPI, blue). Scale bars, 10 μm. **b,** Quantitative comparison of total fluorescence intensity for F-actin, phospho-myosin, vimentin, and DAPI between 5 and 120 min post-seeding in each fibroblast cell line. Data are presented as mean ± s.d. and *n* represents the number of independent cells analyzed. Statistical significance was assessed using the Wilcoxon rank-sum test. *P* values are given in figures, values >0.05 are considered non-significant (n.s.).

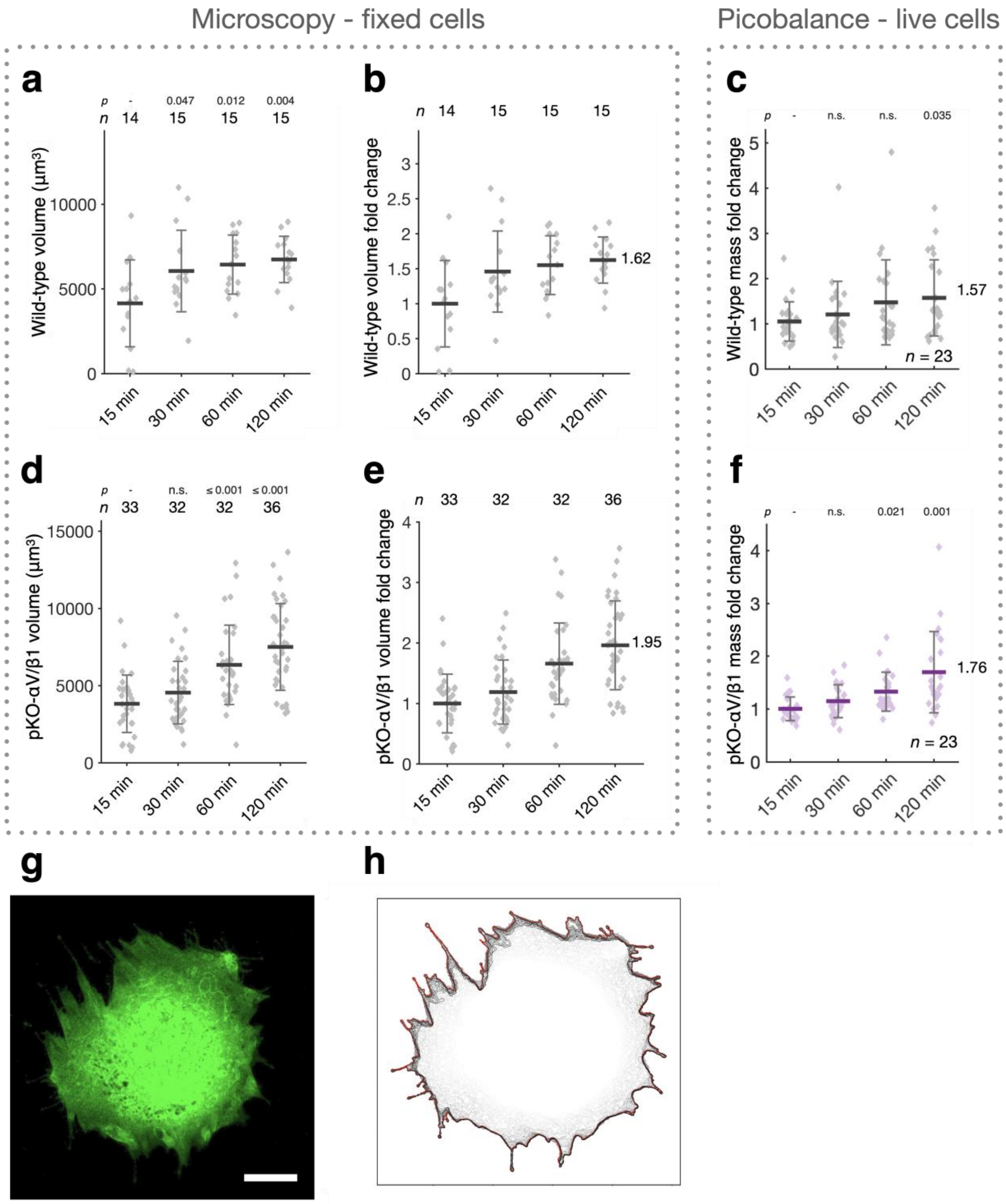

Supplementary Figure S3. Correlation between cell volume and mass measurements. a-b, Quantification of the volume of wild-type fibroblasts on fibronectin-coated 8-well slides 15 min, 30 min, 1 h, and 2 h post-seeding. Volumes determined using CellTracker dye and confocal microscopy are shown in both absolute and relative terms. **c,** Fold change in mass of single wild-type fibroblasts adhering on fibronectin-coated microcantilevers for 2 h. Measurements were performed under cell culture conditions (37°C and 5% CO_2) in DMEM medium supplemented with FBS following overnight starvation. **d-e,** Quantification of pKO-αV/β1 fibroblast volume on fibronectin-coated slides 15 min, 30 min, 1 h, and 2 h post-seeding. Volumes determined using CellTracker dye and confocal microscopy are shown in both absolute and relative terms. **f,** Fold change in mass of single pKO-αV/β1 fibroblasts on fibronectin-coated microcantilevers for 2 h. Measurements were performed under cell culture conditions (37°C and 5% CO_2) in DMEM medium supplemented with FBS following overnight starvation. Data are presented as mean ± *s.d.*, with *n* indicating the number of independent cells

measured. Statistical significance was assessed using the Wilcoxon rank-sum test. **g,** Fluorescent image of a wild-type fibroblast labeled with CellTracker dye, after 2 h of adhesion on a fibronectin-coated slide. Scale bar, 10 μm. **h,** Image segmentation example of the CellTracker-stained fibroblast, processed using custom in-house software to visualize cell volume. *P* values are given in figures, values >0.05 are considered non-significant (n.s.).

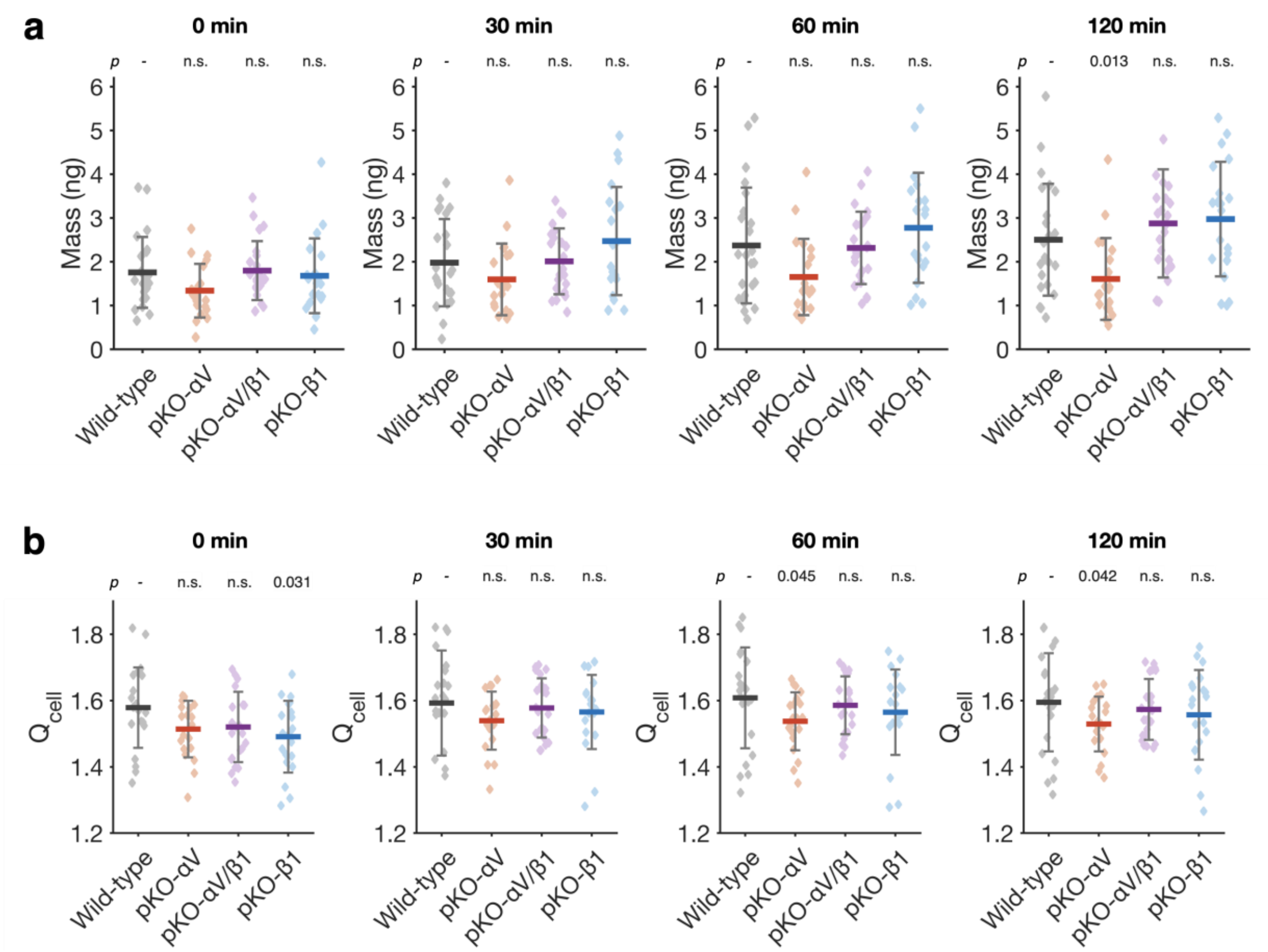

Supplementary Figure S4. Statistical analysis of total mass and Q_{cell} of integrin knockout fibroblast cell lines under physiological conditions. Mass and Q-factor of wild-type, pKO-αV, pKO-αV/β1 and pKO-β1 fibroblasts adhering to fibronectin-coated cantilevers and monitored right after attachment (0 min) and at 30 min, 60 min and 120 min post attachment. Measurements were performed under cell culture conditions (37°C and 5% CO_2) in DMEM medium supplemented with FBS following overnight starvation. Data are presented as mean ± s.d. Statistical significance was assessed using the Wilcoxon rank-sum test. *P* values are given in figures, values >0.05 are considered non-significant (n.s.).

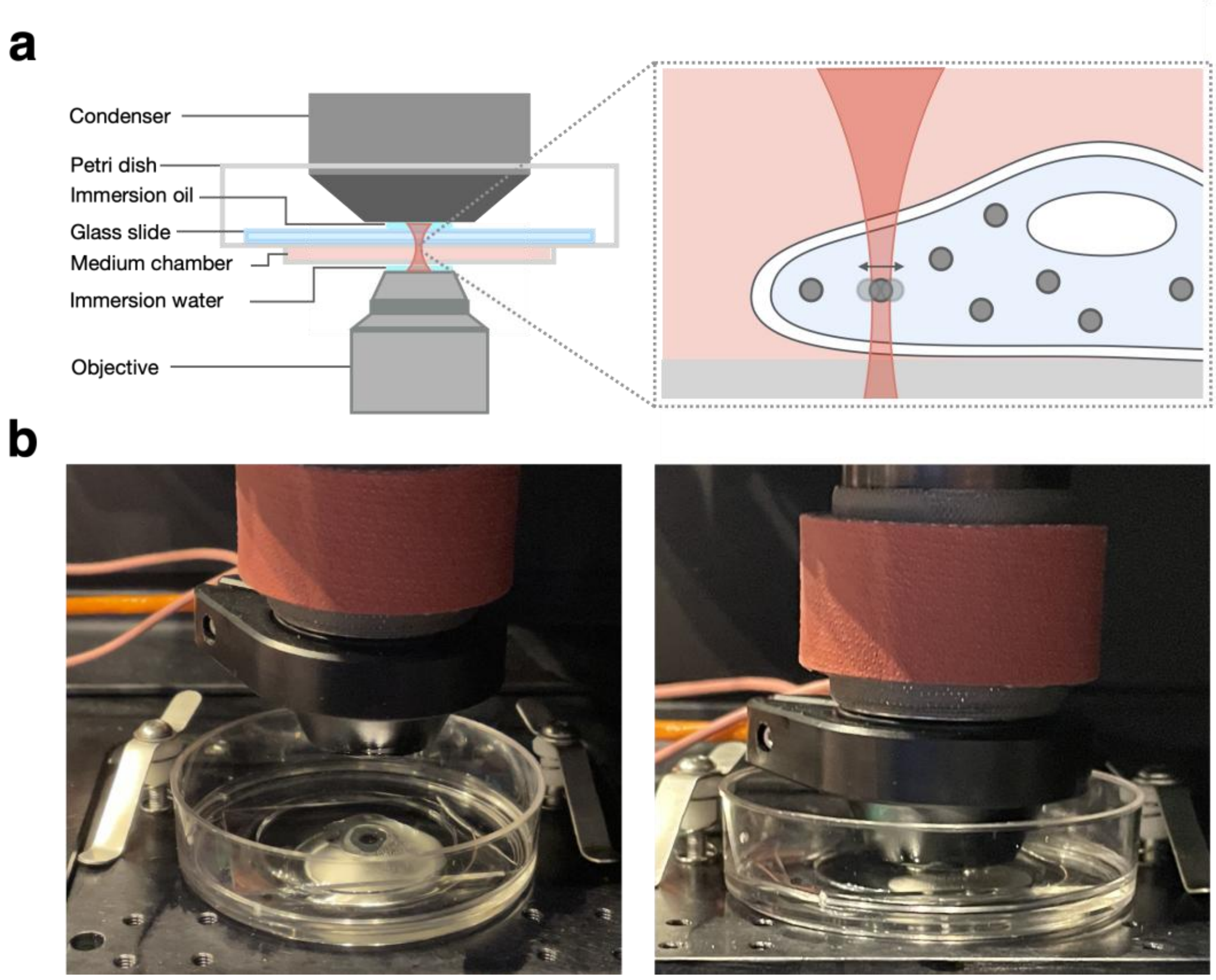

Supplementary Figure S5. Optical tweezers setup for cell microrheology measurements. a, Cells with internalized 0.49 µm carboxyl latex beads were placed on a glass-bottom Petri dish, positioned between a water immersion objective (60x, NA 1.2, Nikon) and an oil immersion condenser (60x, NA 1.4, Leica) in a C-Trap optical tweezer (Lumicks, Netherlands) system. The condenser, positioned above the dish, captured photons from the 1,064 nm infrared trapping laser. A thin glass cover slip separated the condenser from the cell culture medium, with both objective and condenser pre-warmed to 30–35°C. **b,** Photographs of the setup. Condenser position during experiment preparation (left) and in contact with the glass slide during measurements (right).

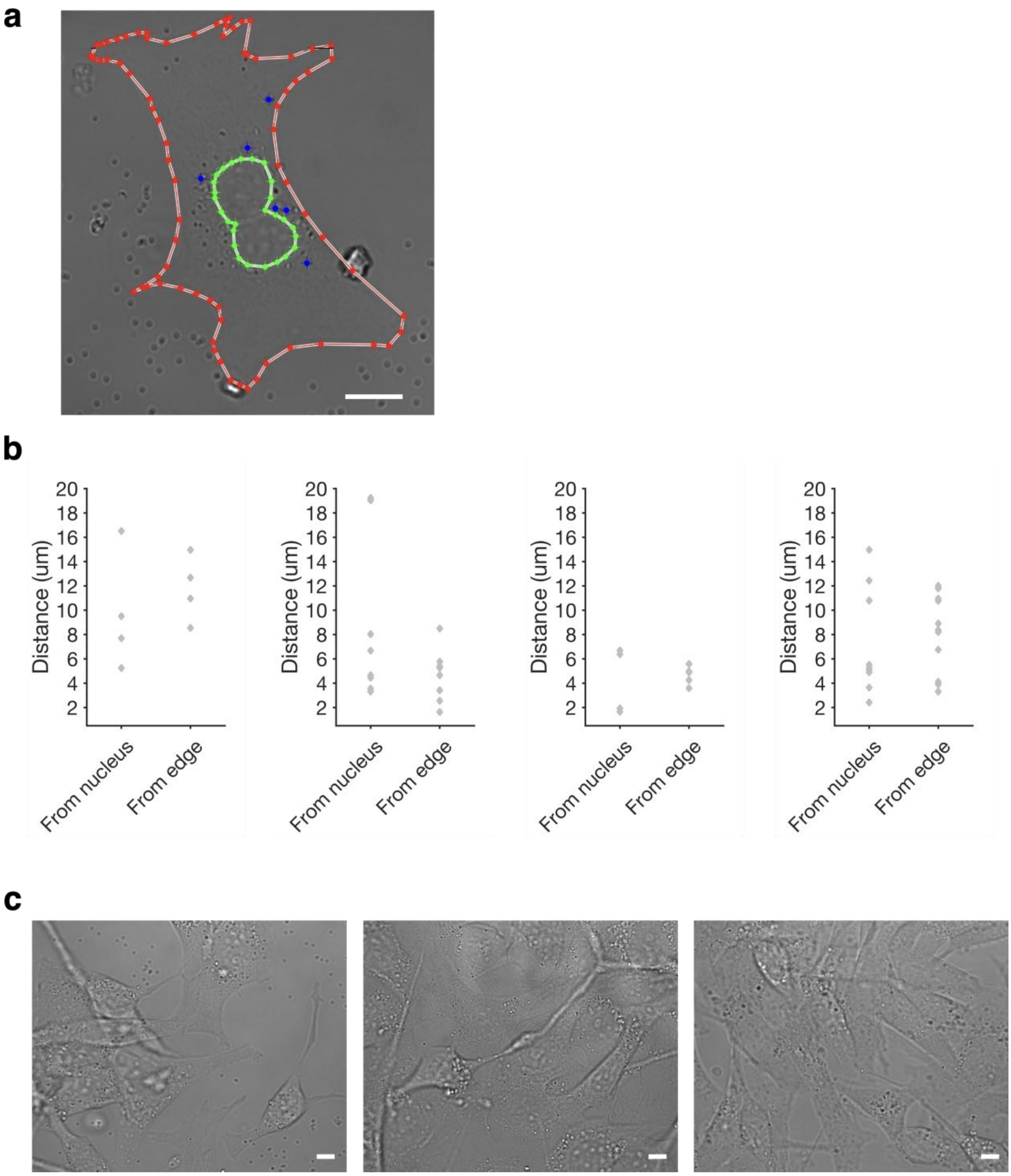

Supplementary Figure S6. Extended optical tweezers data. a, Bright field image of a wild-type fibroblast spread on fibronectin-coated coverslips with endocytosed latex beads. The cell edges are marked in red, the nucleus contour in green and the beads in blue. Scale bar, 10 μm. **b,** Distribution analysis of 0.49 μm carboxylated latex beads endocytosed overnight by wild-type fibroblasts cultured in DMEM supplemented with FBS at 37°C and 5% CO_2.Therepresentativegraphsillustratethespatial distribution of the beads (data points) inside four different fibroblasts, showing their distances from the nucleus and the nearest cell edge. **c,** Bright field images of wild-type fibroblasts incubated with 0.49 μm carboxylated latex for 2 days in DMEM supplemented with FBS at 37°C and 5% CO_2. Scale bars, 10 μm.

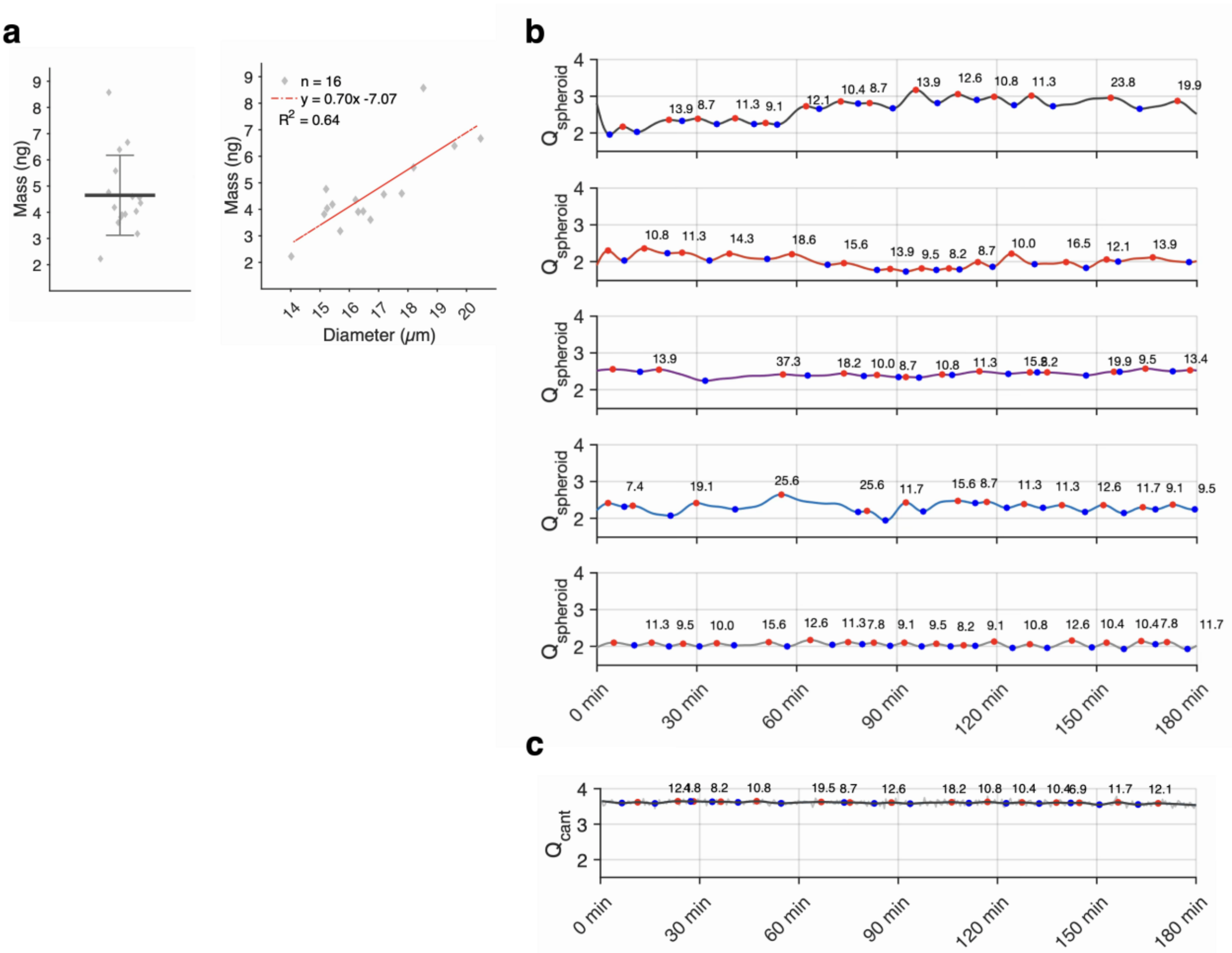

Supplementary Figure S7. Cancer-associated fibroblasts (CAFs) and human spheroids extended data. a, Mass of single primary CAFs from the human rectum as measured directly after attachment to ConA-coated cantilevers. During measurement the cells showed rounded shape and their individual masses could be approximated from their diameter (bright field images). Measurements were conducted in DMEM medium supplemented with FBS at 37°C with 5% CO_2. On the left, data are presented as mean ± *s.d*. On the right, the mass from each single cell is plotted against the diameter of the cell measured by optical microscopy after attachment on the cantilever. *n* indicates the number of independent cells measured. **b-c,** $Q_{spheroid}$ of five individual human spheroids (**b**) and Q_{cant} of a bare cantilever used for the measurements (**c**). The data was smoothed using an 8th-order low-pass filter with a cutoff frequency of 0.1 Hz. Local maxima are indicated by red dots, local minima with blue dots. Time periods in minutes between local maxima are given above the corresponding data points. The Q-factor oscillations of the spheroids are much larger in amplitude compared to that of the bare cantilever, thus suggesting that spheroids increase the oscillation amplitude of the cantilever.

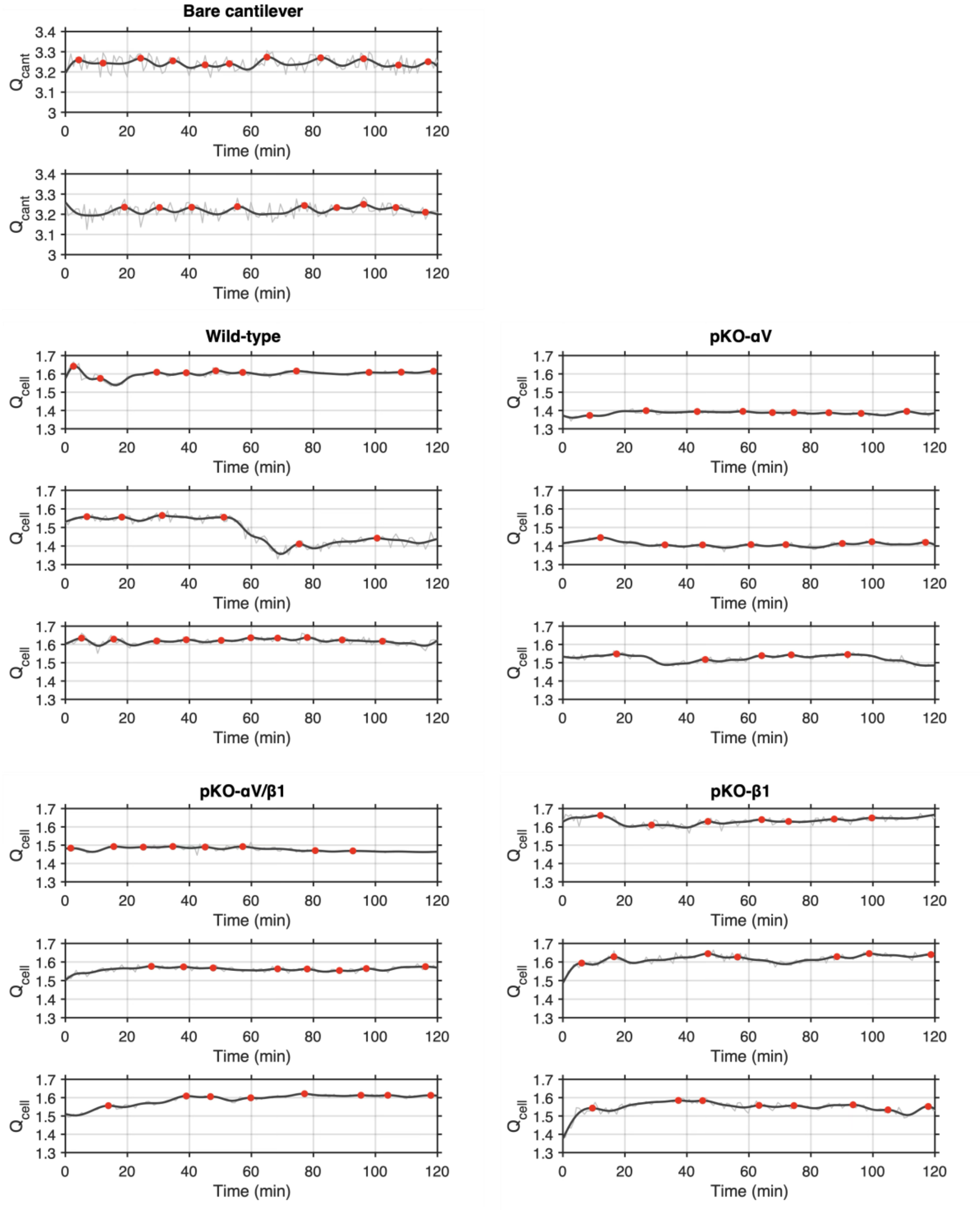

Supplementary Figure S8. Single cell Q_{cell} oscillation analysis of cantilevers without and with fibroblasts attached. Representative curves of Q_{cell} for wild-type, pKO-αV/β1, pKO-αV, and pKO-β1 fibroblasts spreading on a fibronectin-coated cantilever for 2 h, alongside Q_{cant} from a bare fibronectin-coated cantilever without cells. The data was smoothed using an 8th-order low-pass filter (cutoff frequency: 0.1 Hz), and local maxima are indicated by red dots. The average Q-factor oscillation amplitude was 0.036 ± 0.053 for wild-type fibroblasts, 0.017 ± 0.013 for pKO-αV/β1 fibroblasts, 0.019 ± 0.020 for pKO-αV fibroblasts, 0.039 ± 0.064 for pKO-β1 fibroblasts, and 0.033 ± 0.011 for bare cantilevers. The Q-factor oscillations of the single fibroblasts have a comparable amplitude to those of the bare cantilever, suggesting that the fibroblasts do not introduce additional damping to the cantilever.

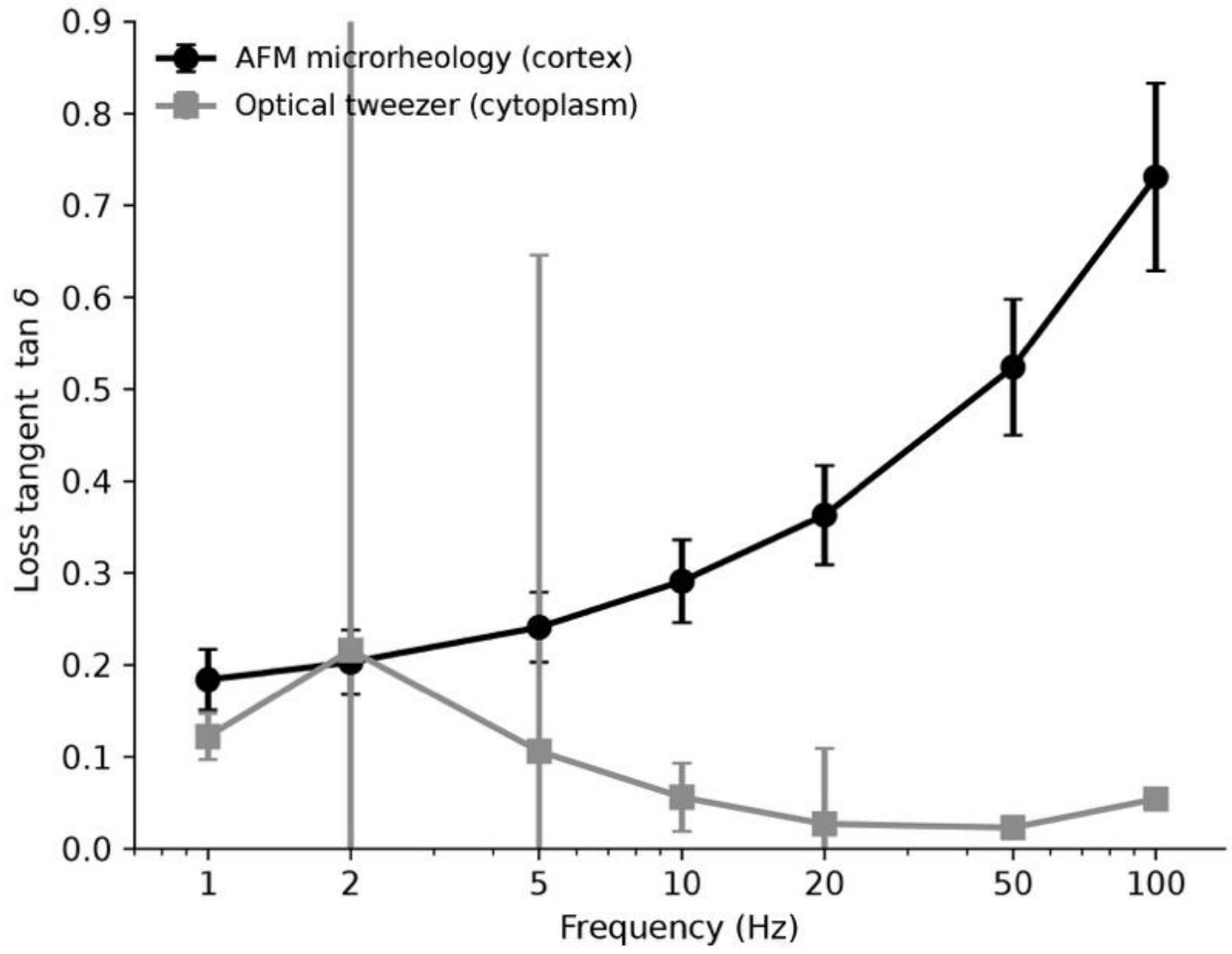

Supplementary Figure S9. Viscoelastic loss tangent tan *δ* calculated from the storage moduli *G'* and loss moduli *G''* of wild-type fibroblasts revealed from optical tweezer and from AFM-based microrheological measurements. *G'* and *G''* values to calculate the loss tangent tan *δ* for the example of wild-type fibroblasts were taken from Figure 4. Values represent the mean, error bars the s.e.m.. The number of wild-type fibroblasts analyzed corresponds to those characterized in Figure 4, *i.e.*, 45 fibroblasts have been characterized by optical tweezers and 56 fibroblasts have been characterized by AFM.
